# Murine myeloid cell MCPIP1 suppresses autoimmunity by regulating B-cell expansion and differentiation

**DOI:** 10.1242/dmm.047589

**Published:** 2021-03-18

**Authors:** Ewelina Dobosz, Georg Lorenz, Andrea Ribeiro, Vivian Würf, Marta Wadowska, Jerzy Kotlinowski, Christoph Schmaderer, Jan Potempa, Mingui Fu, Joanna Koziel, Maciej Lech

**Affiliations:** 1 Department of Microbiology, Faculty of Biochemistry Biophysics and Biotechnology, Jagiellonian University, Krakow 30-387, Poland; 2 Klinikum rechts der Isar, Department of Nephrology, Technical University Munich, Munich 81675, Germany; 3 LMU Klinikum, Medizinische Klinik und Poliklinik IV, Ludwig-Maximilians-Universität München, Munich 80336, Germany; 4 Department of General Biochemistry, Faculty of Biochemistry, Biophysics and Biotechnology, Jagiellonian University, Krakow 30-387, Poland; 5 Department of Oral Immunity and Infectious Diseases, University of Louisville School of Dentistry, University of Louisville, Louisville, KY 40202, USA; 6 Department of Biomedical Science and Shock, Trauma Research Center, School of Medicine, University of Missouri-Kansas City, Kansas City, MO 64108, USA

**Keywords:** Autoimmunity, Myeloid cells, Lupus nephritis, MCPIP1, Autoantibodies

## Abstract

Myeloid-derived cells, in particular macrophages, are increasingly recognized as critical regulators of the balance of immunity and tolerance. However, whether they initiate autoimmune disease or perpetuate disease progression in terms of epiphenomena remains undefined.

Here, we show that depletion of MCPIP1 in macrophages and granulocytes (*Mcpip1^fl/fl^-LysM^cre+^* C57BL/6 mice) is sufficient to trigger severe autoimmune disease. This was evidenced by the expansion of B cells and plasma cells and spontaneous production of autoantibodies, including anti-dsDNA, anti-Smith and anti-histone antibodies. Consequently, we document evidence of severe skin inflammation, pneumonitis and histopathologic evidence of glomerular IgG deposits alongside mesangioproliferative nephritis in 6-month-old mice. These phenomena are related to systemic autoinflammation, which secondarily induces a set of cytokines such as *Baff*, *Il5*, *Il9* and *Cd40L*, affecting adaptive immune responses. Therefore, abnormal macrophage activation is a key factor involved in the loss of immune tolerance.

Overall, we demonstrate that deficiency of MCPIP1 solely in myeloid cells triggers systemic lupus-like autoimmunity and that the control of myeloid cell activation is a crucial checkpoint in the development of systemic autoimmunity.

## Abstract (precis)

**Summary:** Analyses of mice deficient in myeloid MCPIP1 reveal a function of myeloid MCPIP1 in the transition from autoinflammation to autoimmunity and a role for macrophage-dependent immune activation as a trigger of secondary autoimmunity.

## INTRODUCTION

Monocyte chemoattractant protein-1 induced protein-1 (MCPIP1; also known as Regnase-1) displays endoribonuclease activity and regulates deubiquitination (Liang et al., 2010; Matsushita et al., 2009; Mizgalska et al., 2009). The protein, encoded by the *ZC3H12A* gene, is a critical negative regulator of inflammation (Jura et al., 2012; Li et al., 2017; Liu et al., 2016). Its roles in inhibiting various inflammatory processes initiated by pattern recognition receptor (PRR) ligands, as well as regulating chemokines and cytokines such as IL-8, IL-6 and IL-1β, or affecting cytokine signaling pathways, as observed for IL-17, were described previously (Dobosz et al., 2016; Garg et al., 2015; Matsushita et al., 2009; Mizgalska et al., 2009). MCPIP1 restricts Toll-like receptor (TLR) signaling by cleaving target mRNAs (Matsushita et al., 2009) or affecting deubiquitination of molecules within the NF-κB signaling pathway (Liang et al., 2010; Skalniak et al., 2009). Furthermore, MCPIP1 was described as a transcription factor for apoptotic gene families (Zhou et al., 2006). MCPIP1-deficient mice die prematurely and display signs of severe multiorgan inflammation (Liang et al., 2010; Matsushita et al., 2009; Miao et al., 2013). Moreover, they develop hyper-responsiveness upon exposure to some pathogen-associated molecular patterns (Liang et al., 2010; Matsushita et al., 2009). Despite the general agreement that MCPIP1 is a powerful negative regulator of inflammation, its role in cell-specific responses and autoimmunity remains poorly understood.

The most prominent autoimmune theories indicate several initial triggers for autoimmune disorders; for instance, recognition of self-molecules by innate immunity receptors and expansion of autoreactive T and B cells (Thomas, 2001). Central tolerance removes self-reactive T lymphocytes and self-reactive B cells in the thymus and bone marrow, respectively. Moreover, the self-reactive cells that have escaped this selection are counterbalanced in the peripheral tissues (Petersone et al., 2018). Antigen-presenting cells (APCs) perfectly link innate and adaptive immunity. They are responsible for auto-antigen uptake and its presentation to naïve T cells. Indeed, animals deficient in APCs do not develop sufficient mechanisms of defense (i.e. cross-presentation) and develop systemic autoimmune disorder (Birnberg et al., 2008; Ohnmacht et al., 2009). Furthermore, phagocytes regulate the kinetics of necrotic cell clearance to restrict the concentration of potential auto-antigens within the tissue (Fond and Ravichandran, 2016; Mistry and Kaplan, 2017). Finally, yet importantly, they are key regulators of inflammation (Mosser et al., 2021). These necessary checkpoints within innate immune responses limit the production of autoantibodies and immune complex deposition that trigger autoimmune organ damage.

It has long been speculated that innate myeloid cells balance immunity and tolerance. The findings that innate myeloid cells subtypes robustly respond to extra- and intracellular PRR ligands and orchestrate adaptive immune responses support this hypothesis (Jain and Pasare, 2017). Innate immune cells act via adjusting the constellation of co-stimulatory molecules and secretion of polarizing cytokines, chemokines and other soluble molecules. Tolerogenic myeloid cells, which poorly express co-stimulatory molecules such as CD80, CD86 and MHCII, are believed to skew the T cells towards T-regulatory cells (Tregs) (Jonuleit et al., 2000; Raker et al., 2015; Sato et al., 2003). Therefore, besides the cell number, regulatory mechanisms in APCs could be crucial to shape and balance immune activation of these cells.

Given the role of MCPIP1 as a potent suppressor of inflammation, we hypothesized that macrophage/granulocyte-derived MCPIP1 is responsible for systemic homeostasis and prevents autoimmunity. To verify the cell-intrinsic function of MCPIP1 in chronic inflammation, we generated mice lacking MCPIP1 in hematopoietic cells of the myelomonocytic lineage using *LysM*-cre and *Mcpip1*-floxed mice and examined them for signs of spontaneous autoimmunity. Here, we document that MCPIP1 suppresses the development of autoimmunity with features of systemic lupus erythematosus (SLE) and severe nephritis.

## RESULTS

### Macrophage expression of MCPIP1 suppresses autoimmunity-related transcripts

**Fig. 1.**
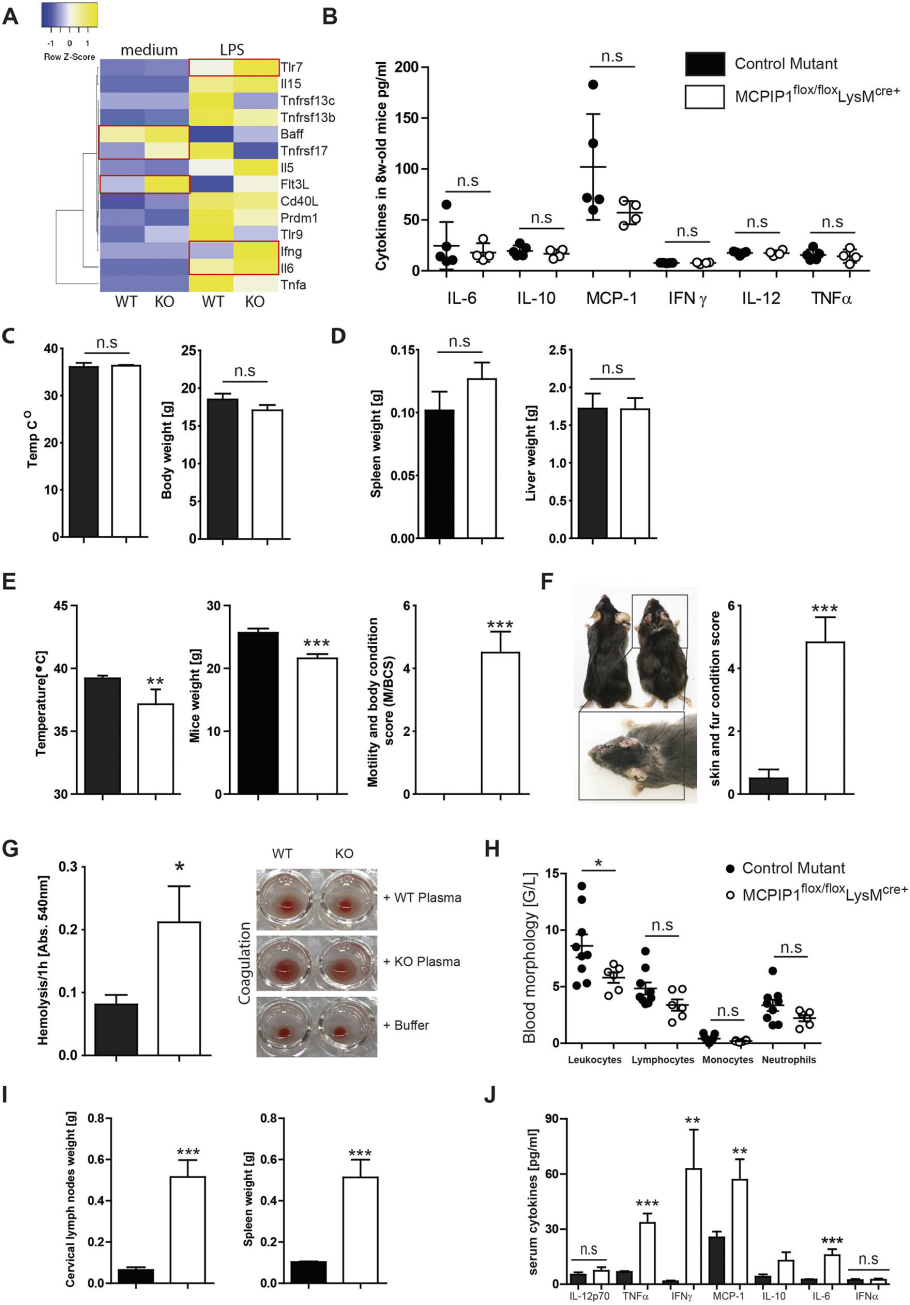
**MCPIP1 suppresses autoinflammation and lymphoproliferation in 6-month-old mice.** (A) Heat map depicting the altered genes from expression analysis of pre-selected genes in bone marrow-derived macrophages isolated from wild type (WT) and MCPIP1 knockouts (KO) and stimulated with 10 ng/ml ultrapure LPS. Red frames mark the genes that were significantly upregulated. (B) Knockout and control mice were bled at 2 months to determine serum levels of IL-6, IL-12, IL-10, IFN-γ, MCP-1 and TNF-α. Data represent means±s.e.m. from at least four mice per group. (C) At 2 months of age, MCPIP1-deficient mice had normal body weight and temperature. Data represent means±s.e.m. from at least four mice per group. (D) At 2 months of age, MCPIP1-deficient mice exhibited no hyperplasia, no lymphoproliferation/splenomegaly and no hepatomegaly. The weights of organs from four mice per group are presented as means±s.e.m. (E) At 6 months of age, MCPIP1-deficient mice displayed decreased body temperature, decreased body weight and increased motility. Data from eight mice per group are presented as means±s.e.m.; **P<0.01, ***P<0.001. (F) At 6 months of age, macrophage-specific MCPIP1-deficient mice exhibited massive skin inflammation. Eight mice per group were scored, and data are presented as means±s.e.m.; ***P<0.001. (G) Macrophage-specific MCPIP1-deficient mice displayed increased hemolysis (means±s.e.m.; *P<0.05) and decreased blood coagulation. (H) Blood morphology revealed a significantly decreased number of leukocytes in knockout mice. Data from at least six mice per group are presented as means±s.e.m.; *P<0.05 (I) At 6 months of age, macrophage-specific MCPIP1-deficient mice exhibited massive hyperplasia of cervical, axillar and mesenteric lymph nodes as well as splenomegaly. The weights of cervical lymph nodes and spleens from 16 mice per group are presented as means±s.e.m.; ***P<0.001. (J) Knockout and control mice were bled at 6 months to determine serum levels of IL-6, IL-12, IL-10, IFN-γ, MCP-1, TNF-α and IFN-α. Data represent means±s.e.m. from at least 16 mice per group; **P<0.01, ***P<0.001. n.s., not significant.

To determine the effect of inflammation on *Mcpip1* expression in primary myeloid cells, we performed real-time PCR analysis of cells stimulated for up to 24 h with lipopolysaccharide (LPS), observing that LPS stimulation rapidly enhances *Mcpip1* gene expression (Fig. S1). We generated macrophage-specific MCPIP1-deficient mice (*Mcpip1^fl/fl^-LysM^cre+^* mice) by crossing the *Mcpip1*-floxed strain with a mouse line expressing cre under the *LysM* (also known as *Lyz2*) promotor (Clausen et al., 1999). We used bone marrow-derived macrophages to assess the gene expression profiles after LPS treatment and investigate the expression of autoimmunity-related transcripts. Several transcripts – such as *Baff* (also known as *Tnfsf13b*), *Tnfrsf17* or *Flt3l* – were highly expressed in knockout bone marrow-derived macrophages. Upon LPS stimulation, we observed increased expression levels of *Tlr7* and proinflammatory cytokines such as *Ifng* and *Il6* (Fig. 1A). First, we hypothesized that the mice would develop a macrophage activation syndrome (MAS) owing to the increased activation of LysM-expressing cells. As primary MAS is a severe complication of rheumatic disease in childhood, which is characterized by natural killer cell defects, uncontrolled activation of macrophages, high fever and a systemic cytokine storm, we investigated a cohort of 8-week-old *Mcpip1^fl/fl^-LysM^cre+^* mice for signs of spontaneous autoinflammation. We did not observe any differences in inflammatory cytokines in the serum (Fig. 1B). Total body weight and temperature did not differ significantly between wild-type and MCPIP1-deficient mice (Fig. 1C). We did not detect any signs of suffering, skin rashes or lesions, splenomegaly and lymphadenopathy at the age of 8 weeks (Fig. 1D). Cumulatively, these data argue against a spontaneous MAS-like phenotype.

### Macrophage expression of MCPIP1 suppresses lymphoproliferation and autoinflammation in 6-month-old mice

Next, we examined the cohort of 6-month-old animals and focused our attention on delayed episodes of the unique clinical syndrome. However, we could not detect high fever, obvious signs of rheumatoid disease or other characteristics of MAS. Instead, we observed significantly reduced body temperature in macrophage-MCPIP1-deficient mice (Fig. 1E). Moreover, macrophage-MCPIP1-deficient mice displayed severe wasting syndrome, as indicated by decreased body weight (Fig. 1E). Besides weight loss, mice displayed other signs of suffering, such as apathy and decreased sensitivity to handling (Fig. 1E). Moreover, knockout mice developed asymmetrical skin rashes in the malar region and skin lesions with eczematous phenotype occurring mainly on the back, ears and eyelids (Fig. 1F). To avoid further discomfort, and to follow ethical standards, we euthanized all animals at 6 months of age at the latest. The analysis of blood revealed further abnormalities in knockouts, including increased hemolysis and blood coagulation (Fig. 1G). Moreover, we observed significant leukopenia in the blood of macrophage-MCPIP-deficient mice (Fig. 1H). Further, the lack of MCPIP1 caused massive splenomegaly and lymphadenopathy at 6 months of age (Fig. 1I). Plasma levels of multiple pro-inflammatory cytokines revealed increased levels of IL-6, TNF-α (also known as TNF), MCP-1 (also known as CCL2) and IFN-γ (Fig. 1J). The two latter findings supported previously published observations (Li et al., 2017). Surprisingly, IFN-α, which plays a predominant role in autoimmune diseases such as SLE, did not differ in plasma between knockouts and controls (Fig. 1J).

**Fig. 2.**
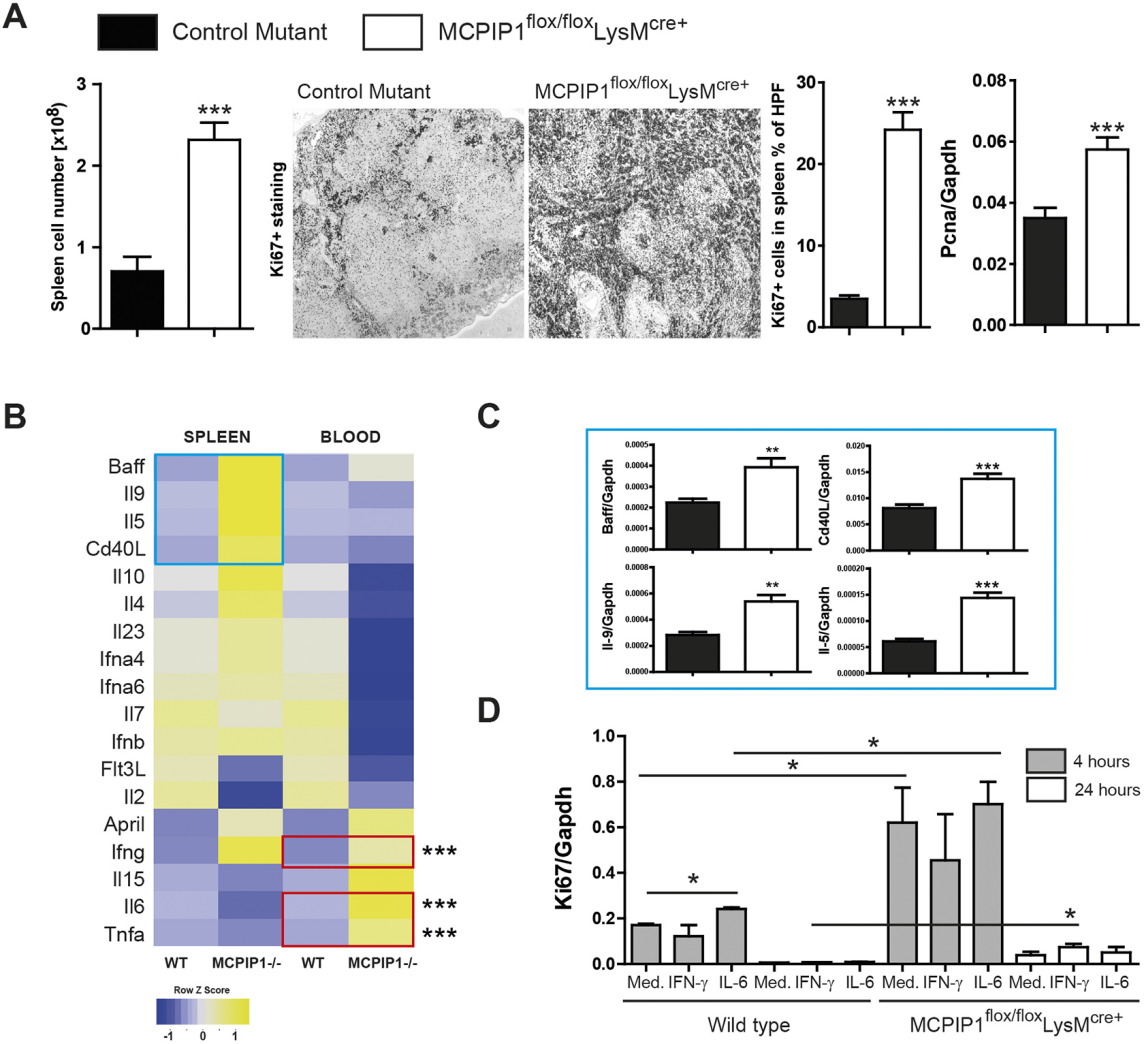
**MCPIP1 regulates the proliferation of splenocytes.** (A) Splenocytes were quantified by flow cytometry; spleen sections were stained with Ki67 proliferation marker and quantified using image software as described in the Materials and Methods (magnification ×100). RNA was isolated from knockout and control mice for real-time PCR analysis. Data are expressed as means of the ratio of *Pcna* versus that of *Gapdh* mRNA. Data are means±s.e.m. from at least eight mice per group; ***P<0.001 versus control mice. (B) Heat map depicting the splenic and whole-blood expression of pre-selected genes of wild type and MCPIP1 knockouts. Red frames mark the genes that were significantly upregulated in the blood; blue frames mark the genes that were considerably changed in the spleen. (C) Genes that were considerably changed in the spleen were investigated in a larger sample (eight wild type versus eight knockouts). (D) Freshly isolated splenocytes were stimulated with selected cytokines to investigate their potential role in splenomegaly. Expression levels of transcripts were quantified by real-time PCR. Data are shown as means of the ratio of the specific mRNA versus that of *Gapdh* mRNA; *P<0.05, **P<0.01, ***P<0.001.

As the spleen is an important component of the immune system, serving as a reservoir for immune cells, regulating red blood cell numbers and generating antibodies, we examined its architecture and content. We observed increased total numbers of spleen cells quantified by flow cytometry. This result was consistent with enhanced Ki-67 (also known as Mki67) staining, indicating a massive lymphoproliferation (Fig. 2A). We confirmed the finding by testing the expression of proliferation marker *Pcna* (Fig. 2A). Further, we analyzed relevant genes involved in autoinflammation to provide an inflammatory/autoimmune profile linked to pathophysiological changes associated with splenomegaly in MCPIP1-deficient mice (Fig. 2B). Whereas mRNA findings from whole-blood samples recapitulate the pro-inflammatory cytokine profile of elevated IL-6, TNFα, and IFN-γ, we detected upregulation of markers linked to the adaptive immune response in lymphoid organs, i.e. *Baff*, *Il9*, *Il5* and *Cd40L* (also known as *Cd40lg*) (Fig. 2B,C). We did not observe any differences in gene expression in the thymus or bone marrow (Fig. S2). Next, we used IL-6 and IFN-γ (significantly upregulated in blood transcriptomic experiment) to examine the cytokine-dependent proliferation of lymphoid cells. Based on the proliferative properties of these cytokines, we hypothesized that the cytokines alone can be responsible for the splenomegaly. The heterogeneous lymphoid-organ-derived cell population from the macrophage-MCPIP1-knockout mice intrinsically showed higher expression of proliferation markers such as Ki67. Stimulation with IFN-γ or IL-6 had a minor effect on the proliferation *in vitro*, indicating that other factors are involved in this process (Fig. 2D). Thus, MCPIP1 deficiency in macrophages favors lymphoproliferation and splenomegaly, and leads to enhanced expression of genes involved in cell proliferation and adaptive immunity regulation.

### MCPIP1 suppress the activation of APCs and controls expansion of B cells and antigen-secreting plasma cells

**Fig. 3.**
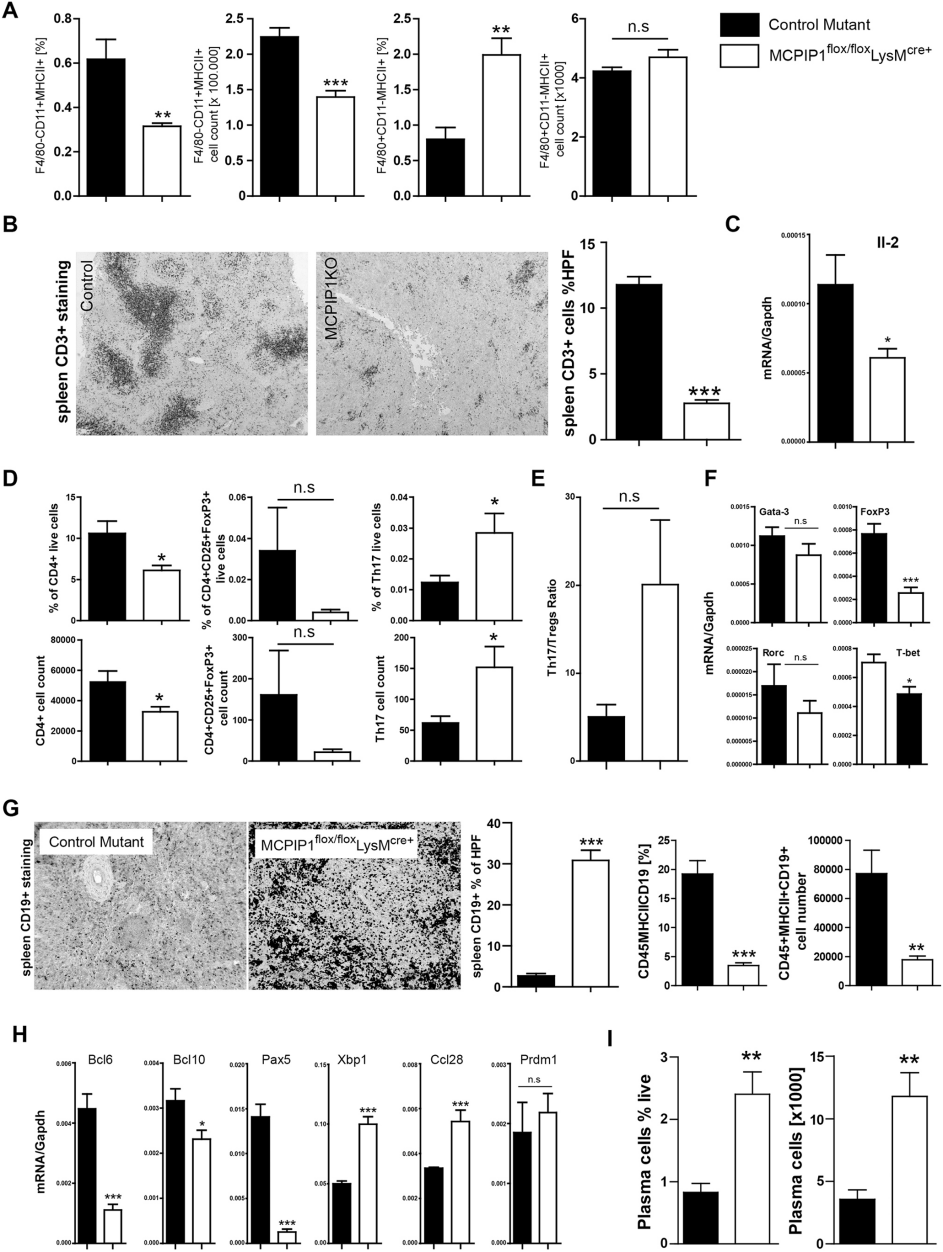
**MCPIP1 suppresses the activation of macrophages and, indirectly, Th17 lymphocytes and B-cell subsets.** (A) The total number of CD11c^+^ dendritic cells and F4/80^+^ macrophages and their activation in spleens was quantified by flow cytometry in both investigated genotypes as described in the Materials and Methods. Data represent means±s.e.m. from at least eight mice per group. **P<0.01, ***P<0.001 versus control mice. (B) Spleen sections were stained for CD3^+^ T cells and quantified with image software as described in the Materials and Methods (magnification ×100). Data are means±s.e.m. from at least eight mice per group; ***P<0.001. RNA was isolated from spleens, and mRNA levels in spleen were analyzed for relevant transcripts. (C) Expression levels of *Il2* were quantified by real-time PCR. Data are shown as means of the ratio of the specific mRNA versus that of *Gapdh* mRNA; *P<0.05. (D,E) The total numbers of the CD4^+^ T-cell subset (D) and Th17/Treg ratio (E) were quantified by flow cytometry in both investigated genotypes as described in the Materials and Methods. Data represent means±s.e.m. from at least eight mice per group. *P<0.05 versus control mice. (F) mRNA levels of T-cell-relevant transcription factors in spleens were quantified by real-time PCR. Data are shown as means of the ratio of the specific mRNA versus that of *Gapdh* mRNA; *P<0.05, ***P<0.001 versus control mice. (G) Spleen sections were stained for CD19^+^ B cells and quantified with image software as described in the Materials and Methods (magnification ×200). Data are means±s.e.m. from at least eight mice per group; ***P<0.001. The total numbers of spleen B220^+^/CD19^+^/MHCII^+^ cells were quantified in 6-month-old mice by flow cytometry. Data are means±s.e.m. of at least eight mice per group; **P<0.01, ***P<0.001 versus control mice. (H) mRNA levels in the spleen were analyzed for B-cell survival and stimulatory factors. Expression levels were quantified by real-time PCR. Data are shown as means of the ratio of the specific mRNA versus that of *Gapdh* mRNA; *P<0.05, ***P<0.001. (I) Mature B cells and kappa light chain^+^ CD138^+^ (also known as Sdc1^+^) plasma cells were quantified in 6-month-old mice by flow cytometry. Data are means±s.e.m. of at least eight mice per group; **P<0.01 versus control mice. n.s., not significant.

Flow cytometry of the spleen revealed an expansion of activated (MHCII^+^) F4/80^+^ cells. Surprisingly, the percentage and number of activated (MHCII^+^) CD11c^+^ (also known as Itgax^+^) dendritic cells decreased in macrophage-specific knockout mice (Fig. 3A). This may indicate dysregulation of immunity as the activation of the particular pathways in CD11c^high^MHCII^+^ dendritic cells orchestrates a balance between inflammatory and regulatory responses (Mencarelli et al., 2018).

How does the activation of APCs in MCPIP1-deficient mice affect lymphocyte subsets? T cells make up the greatest proportion of lymphocytes, and their number, as well as activation status, can affect the systemic cytokine pattern. Therefore, we characterized the T-lymphocyte population in MCPIP1-deficient and control mice. Surprisingly, we observed significantly reduced numbers of T lymphocytes in spleens of MCPIP1-deficient mice, as evidenced by histology and flow cytometry (Fig. 3B). These results were supported by the low expression of the crucial T-cell growth factor *Il2* in the knockout animals (Fig. 3C). Importantly, the phenomenon of lymphopenia was described in patients suffering from an autoimmune disorder in acute phases of the disease. Some patients with SLE display low numbers of lymphocytes and, at the same time, suffer from accelerated anti-self T-cell proliferation (Merayo-Chalico et al., 2016; Theofilopoulos et al., 2001). Interestingly, only one population of T cells, Th17 cells, which are critical for the development of an autoimmune disease, did increase in myeloid-MCPIP1-deficient mice (Fig. 3D). In contrast, other subsets that have strong implications for autoimmunity – namely, regulatory CD4^+^CD25^+^FoxP3^+^ Tregs – were reduced in knockout mice (Fig. 3D). Reduced expression of Flt3L in the lymphoid organs of the knockouts could additionally participate in decreased Treg number (Svensson et al., 2013). Consistently, the mRNA expression of T-cell transcription factors [*Foxp3*, *T-bet* (also known as *Tbx21*)] was also significantly lower compared with that in control animals (Fig. 3F). Thus, MCPIP1 deficiency in macrophages causes significant downregulation of Treg proliferation and skews the Treg/Th17 ratio towards a predominance of Th17 T cells (Fig. 3E).

**Fig. 4.**
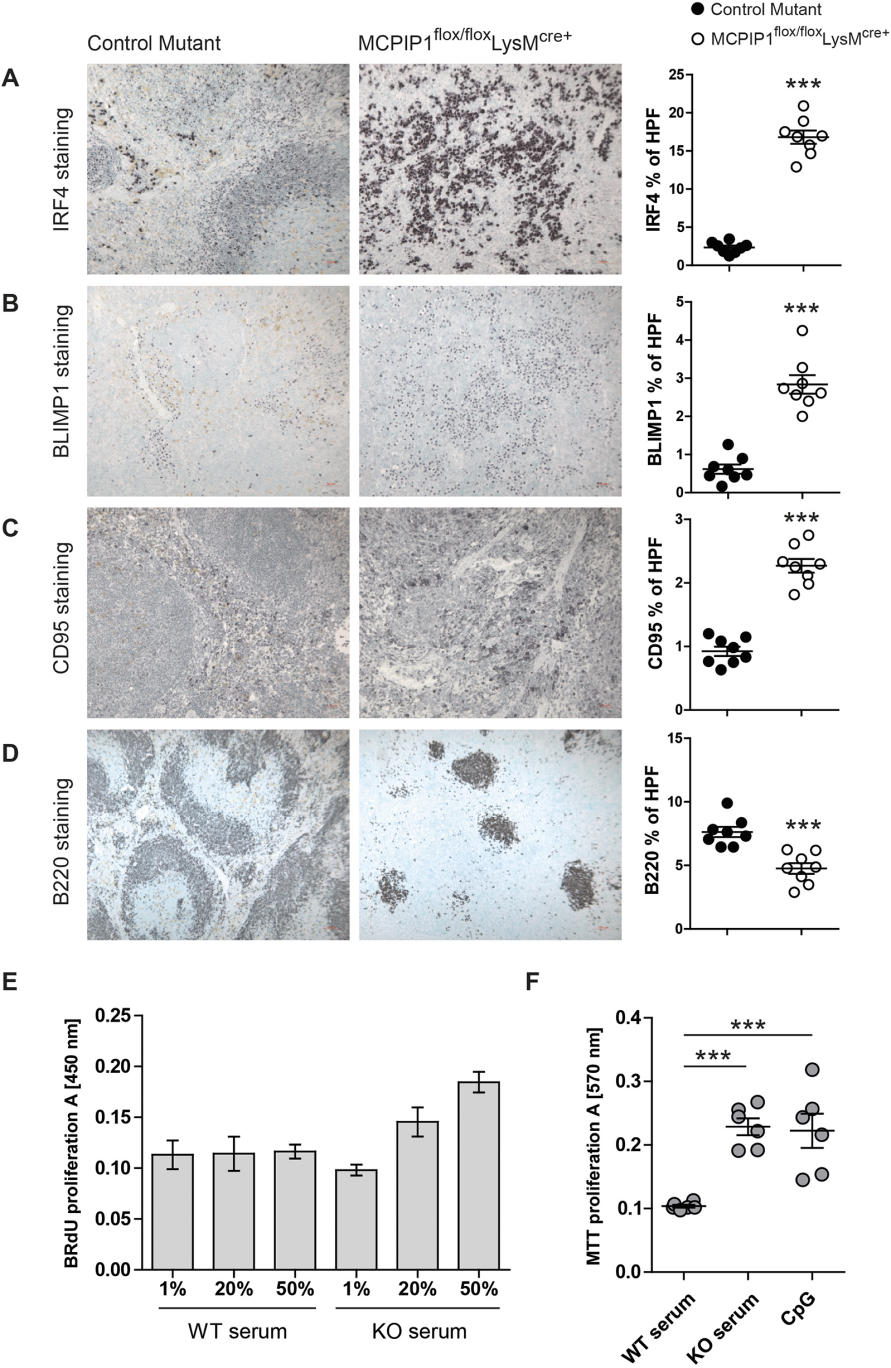
**Macrophage-derived MCPIP1 regulates B-lymphocyte expansion in lymphoid organs.** (A-D) Spleen sections were stained with anti-mouse IRF4, BLIMP1, CD95 and B220 antibodies and quantified by Adobe Photoshop software as percentage of positively stained high-power field (HPF) from eight mice per group; ***P<0.001. (magnification ×200, A,B; ×400, C; ×100, D) (E) The proliferative activity of primary B lymphocytes stimulated with serum from wild-type and knockout mice. Data are means±s.e.m. (F) The proliferative activity of A20 B-lymphocyte cell line stimulated with serum from wild-type and knockout mice and CpG. Data are means±s.e.m.; ***P<0.001.

Surprisingly, despite reduced T-cell population, MCPIP1 deficiency was associated with increased numbers of CD19^+^ B cells (Fig. 3G). The count and percentage of activated MHCII^+^CD19^+^ cells were decreased in MCPIP1-deficient mice. Additionally, we observed decreased mRNA expression of the B-cell inhibitor *Bcl6* in the spleen (Fig. 3H), which prevents plasmablast differentiation. Decreased expression of *Bcl10* and *Pax5*, and enhanced levels of *Xbp1* and *Ccl28* transcripts, implies that myeloid cell-derived MCPIP1 has a strong immunosuppressive effect on plasma cells (Fig. 3H). In line with that, the number of plasma cells was increased in the spleens of knockout mice (Fig. 3I). Next, we stained and quantified the several factors that participate in B-cell maturation in the spleen. We observed a significantly increased number of IRF4^+^, BLIMP1^+^ (also known as PRDM^+^) and CD95^+^ (also known as Fas^+^) cells, but a low level of B220^+^ (also known as PTPRC^+^) cells, in the spleen of the knockout mice (Fig. 4A-C). The knockouts revealed abnormal germinal center (GC) formation, characterized by an irregular pattern of B220 staining (Fig. 4D). Furthermore, both freshly isolated B cells and a B-lymphocyte cell line (A20) showed enhanced proliferative activity when stimulated with serum from knockout mice (Fig. 4E,F). Thus, MCPIP1 has a strong suppressive effect on B-lymphocyte differentiation and plasma cell maturation.

### Macrophage expression of MCPIP1 suppresses lupus autoantibody production in mice

**Fig. 5.**
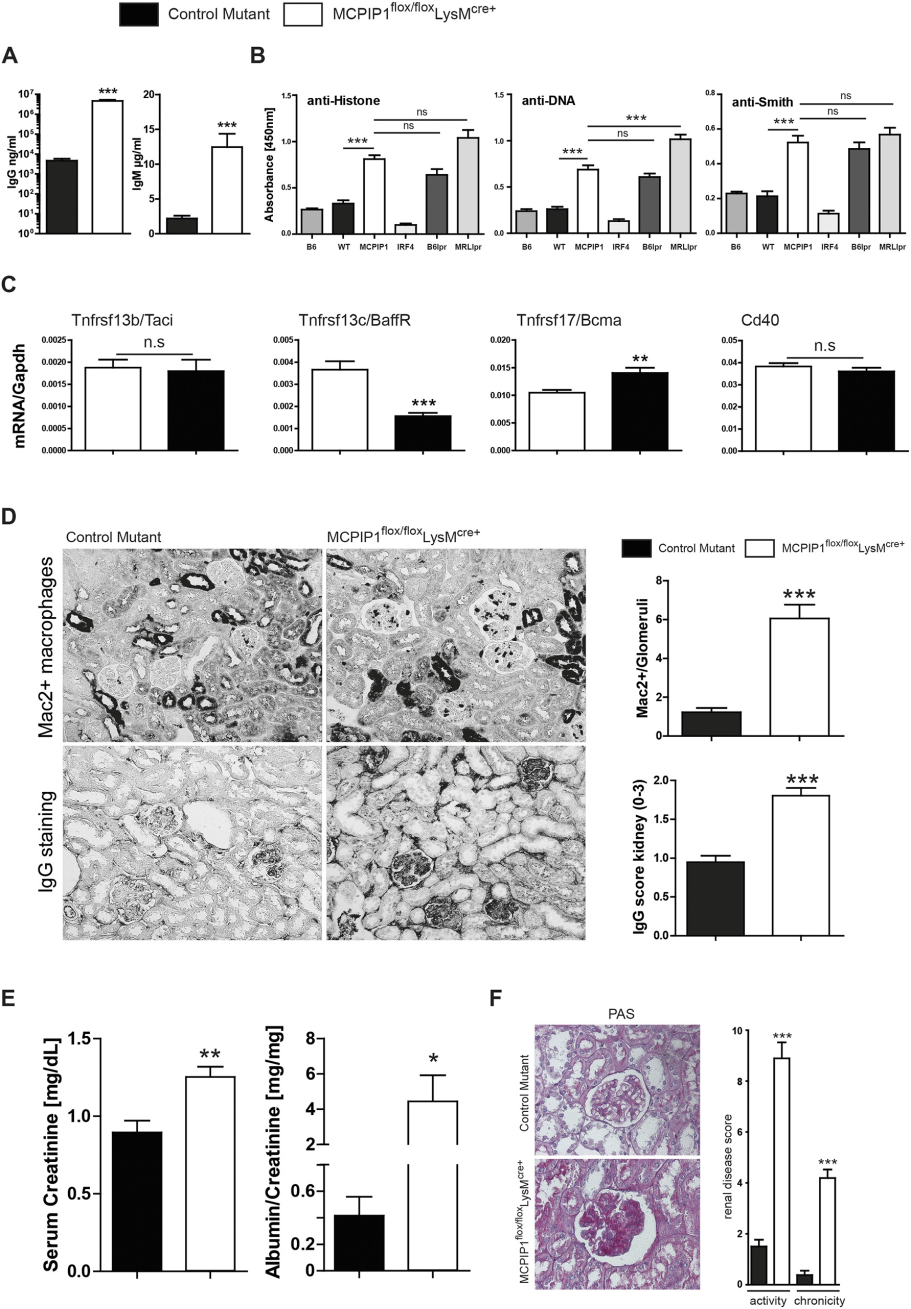
**Macrophage-derived MCPIP1 limits antibody production and kidney injury.** (A,B) Mice from both groups were bled at 6 months to determine serum levels of IgG and IgM (A), as well as anti-histone, anti-dsDNA and anti-Smith autoantibodies (B) by ELISA. Data show means±s.e.m. from at least 16 mice per group; ***P<0.001 versus control mice. (C) mRNA levels in the spleen were analyzed for B-cell-relevant genes. Expression levels were quantified by real-time PCR. Data are shown as means of the ratio of the specific mRNA versus that of *Gapdh* mRNA; **P<0.01, ***P<0.001. (D) Kidney sections were stained for Mac2 and total IgG, and quantified by counting (Mac2) or by using a semi-quantitative score (IgG) (magnification ×400). Data are shown as means of at least 40 scored HPFs or quantified in glomeruli from at least eight mice per group; ***P<0.001. (E) Kidney parameters were measured in both groups of mice. Data are means±s.e.m. of eight to 16 mice per group; *P<0.05, **P<0.01 versus control mice. (F) Kidney sections were stained with periodic acid–Schiff (PAS) and Masson Trichrome, and quantified using a semi-quantitative score (magnification ×400). Data are shown as means of at least 40 scored HPFs or quantified in glomeruli from at least eight mice per group; ***P<0.001. n.s., not significant.

Consequently, we wondered whether myeloid cell MCPIP1 deficiency would enhance IgG and autoantibody production. As expected, plasma IgG and IgM levels were significantly increased in knockout mice, supporting our flow cytometry finding (Fig. 5A). Owing to leukopenia, hemolysis, skin abnormalities, a Th17-prone T-cell signature and the presence of lymphoproliferation, we suspected lupus-like disease as the underlying condition in myeloid-cell-MCPIP1-deficient animals (Raymond et al., 2017). Thus, we screened for characteristic autoantibodies. Among those, anti-double-stranded DNA (dsDNA), anti-Smith and anti-histone IgG were significantly increased in macrophage-specific MCPIP1-knockout mice (Fig. 5B). Moreover, we investigated the splenic mRNA expression of receptors relevant for adaptive immune responses such as *Tnfsrf13b* (encoding transmembrane activator and calcium modulator ligand interactor Taci), *Tnfrsf13c* (Baff receptor), *Tnfsrf17* [B-cell maturation antigen (*Bcma*)] and *Cd40*. We observed significant upregulation of transcript encoding BCMA, which is induced in late-memory B cells. BCMA is highly expressed in cells that undergo antibody-producing differentiation and is present on all plasma cells (Benson et al., 2008; Mackay et al., 2003; O'Connor et al., 2004). It seems to be important for the survival of long-lived plasma cells (O'Connor et al., 2004; Xu and Lam, 2001). During plasma cell differentiation, the expression of Baff receptor is decreased (O'Connor et al., 2004). We observed this expression pattern in MCPIP1 mutants compared to controls (Fig. 5C). Thus, MCPIP1 has a strong suppressive effect on autoantibody production.

### Macrophage MCPIP1 suppresses renal inflammation and lupus nephritis in mice

SLE in human patients is frequently associated with glomerulonephritis (Lech and Anders, 2013; Pan et al., 2019). All macrophage-specific knockouts spontaneously developed renal inflammation evidenced by massive infiltration of immune cells (Fig. S3). This histopathological finding suggests strong inflammatory responses in both interstitial and glomerular compartments of the kidney. Furthermore, Mac2^+^ macrophage numbers inside the glomerular tufts and in glomerular IgG deposits were significantly increased in the knockout mice (Fig. 5D). Renal functional parameters, such as serum creatinine, increased in MCPIP1-deficient mice (Fig. 5E). Furthermore, proteinuria/albuminuria levels were elevated in knockout mice (Fig. 5E). Histopathological analyses of periodic acid–Schiff (PAS)-stained samples revealed mesangioproliferative glomerulonephritis characterized by an increased number of mesangial cells and mesangial matrix expansion (increased glomerular tuft area) in the glomeruli in kidneys of *Mcpip1^fl/fl^-LysM^cre+^* mice (Fig. S4). WT-1 staining was analyzed separately to obtain the number of podocytes per glomerulus. In contrast to the renal tissues of knockout mice, renal tissues of control mice broadly expressed the podocyte marker WT-1, which was exclusively detected along the glomerular structures (Fig. S4). The composite activity and chronicity score for lupus nephritis was significantly increased in *Mcpip1^fl/fl^-LysM^cre+^* mice (Fig. 5F). Together, these results indicate that lack of MCPIP1 in macrophage-like cell types induces severe lupus-like immune complex nephritis, as control mice displayed no such phenotype. Despite normal B- and T-cell genetics, myeloid cell MCPIP1 deficiency is therefore sufficient to trigger classical features of murine SLE and lupus nephritis. These data emphasize an indispensable role for myeloid cells in balancing tolerance versus autoimmunity.

### The autoimmunosuppressive effect of MCPIP1 relates to the regulation of proinflammatory cytokines

**Fig. 6.**
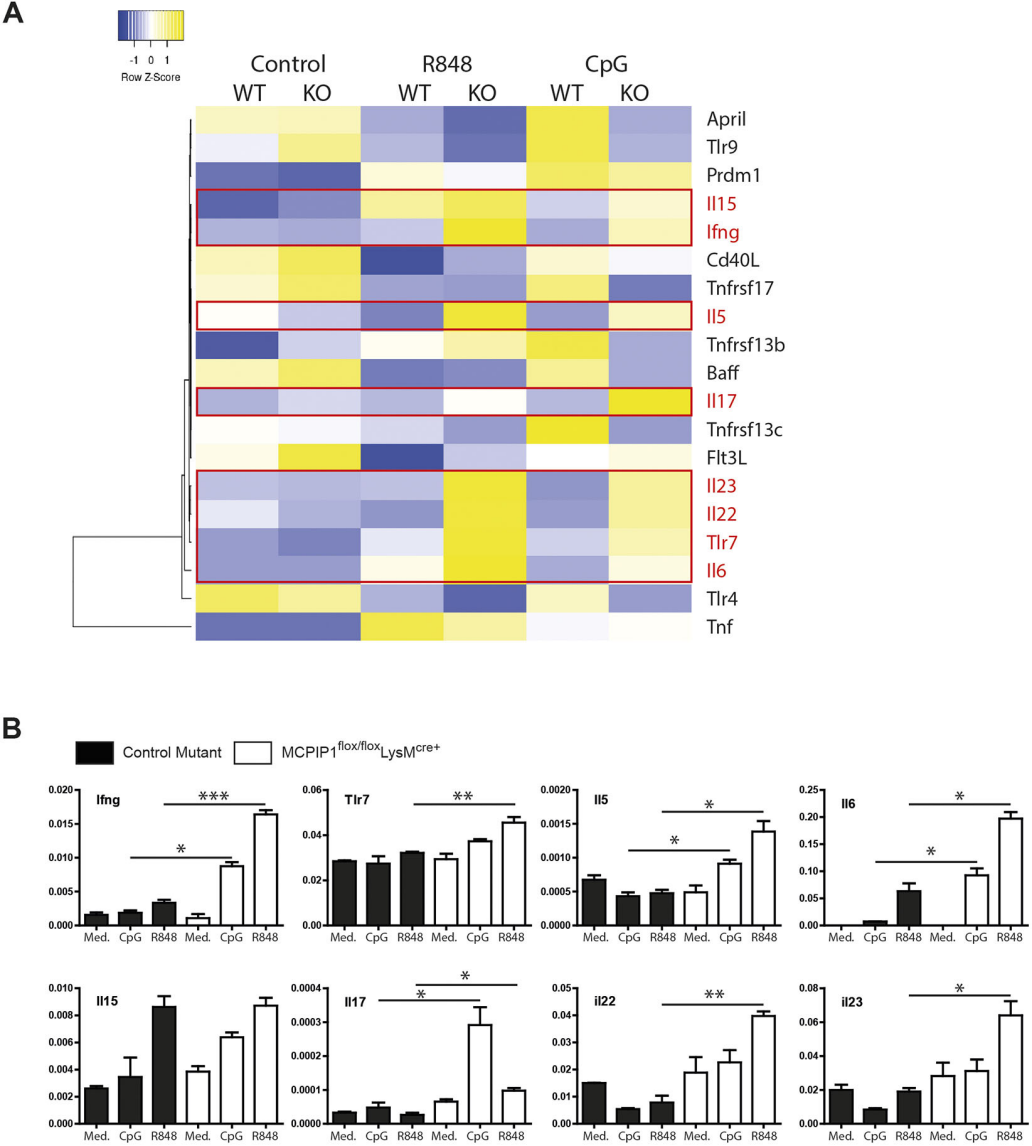
**MCPIP1 orchestrates autoimmunity by affecting TLR7 and TLR9 signaling.** (A) Heat map depicting the altered genes from expression analysis of pre-selected genes in bone marrow-derived macrophages isolated from wild type and MCPIP1 knockouts and stimulated with R848 and CpG. Red marks the genes that were upregulated in knockouts upon stimulation. (B) Expression of selected genes was determined in bone marrow-derived macrophages 4 h post-stimulation with R848 and CpG. Data represent means±s.e.m.; *P<0.05, **P<0.01, ***P<0.001.

First, to elucidate the phenotype of the myeloid-*MCPIP1*-deficient mice, we tested the expression of interferons and interferon-dependent genes in lymphoid organs and blood. We did not observe any significant increase in *Mx1*, *Ifit1*, *Ifit3* or interferons in knockout mice (Fig. S5). Second, because the observed phenotype is routed in aberrant myeloid cell function, which secondarily stimulates B- and plasma-cell autoimmunity, and because macrophages of young MCPIP1-deficient mice displayed inducible overexpression of TLR-7 (Fig. 1), we speculated that macrophages would upregulate B-cell-activating factors upon stimulation with TLR-7 (R848) and TLR-9 (CpG) ligands. Herein, we found significant upregulation of *Tlr7*, *Ifng*, *Il5*, *Il6*, *Il15*, *Il17*, *Il22* and *Il23* (Fig. 6A). Data were replicated by real-time PCR, with similar results except for *Il15* (Fig. 6B). Direct binding and mRNA suppression by MCPIP1 has only been reported for *Il6* and not the remaining dysregulated transcripts (Mino et al., 2015).

In conclusion, MCPIP1 deficiency solely in macrophages causes hyper-responsiveness of macrophages to endogenous ligands, i.e. to TLR-7. In turn, MCPIP1-deficient macrophages produce increased transcripts of IL-17-stimulating cytokines, such as *Il23*, *Il22* and *Ifng*, which favor pathologies associated with adaptive immune response. These data offer a potential explanation for how macrophage-dependent hyperinflammation causes a break in tolerance and secondary expansion of autoreactive plasma cells.

## DISCUSSION

MCPIP1 is a member of a CCCH zinc-finger-containing protein family, and *MCPIP1* was identified as an MCP-1-induced gene in human monocytes (Zhou et al., 2006). It was described as a negative regulator of proinflammatory activation of macrophages *in vitro* (Liang et al., 2008). However, its cell-specific role in the regulation of innate and adaptive immune responses is still unclear. Using *Mcpip1^fl/fl^-LysM^cre+^* mice, we have identified a crucial role for macrophage/granulocytes in maintaining immune homeostasis (Clausen et al., 1999). Our study has also revealed an important role for macrophage-dependent innate immune activation as a trigger of secondary autoimmunity, despite the genetic integrity of T and B cells. The experimental *Mcpip1^fl/fl^-LysM^cre+^* mice demonstrated that some features of autoimmune disease, such as autoantibodies and mesangioproliferative glomerulonephritis, are typical of SLE, whereas others, like lymphadenopathy, splenomegaly and dysregulation of lymphocyte activation, can be linked to autoimmune lymphoproliferative syndrome.

Current understanding of the effects of macrophages in certain autoimmune diseases is limited. Macrophages have been suspected to play a role in regulating self-tolerance, whereas B- and T-cellular adaptive immunity is considered to be more important (Navegantes et al., 2017). On the other hand, Lyn-deficient mice were shown to develop lupus-like autoimmunity and nephritis in a dendritic cell MyD88-dependent manner, indicating that TLR-dependent hyperinflammation can trigger secondary autoimmunity (Lamagna et al., 2013). Similarly, macrophages are well recognized for their abilities to change their phenotype and regulate immune responses (Navegantes et al., 2017). Therefore, the phenotypic and activation properties of macrophages lacking regulatory mechanisms could lead to the identification of more effective therapeutic strategies in the future. We showed that MCPIP1 expression in macrophage/granulocytes (*LysM^+^* cells) protects against spontaneous lupus-like autoimmunity. This was evidenced by the fact that several B-cell differentiation and survival factors/cytokines were de-regulated, promoting the expansion of plasma cells and the production of autoantibodies. We showed that the deletion leads to the expansion of IRF4^+^, BLIMP1^+^ and CD95^+^ cells in lymphoid organs. IRF4 controls several events of B-lymphocyte development and maturation, including pre-B-cell differentiation, marginal zone B-cell development and plasma cell differentiation (Ma et al., 2008; Sciammas et al., 2006; Simonetti et al., 2013). As GCs display a zone of collaboration between proliferating B cells, T-helper cells and dendritic cells, the abnormal GC formation observed in the spleen of knockout mice indicates homeostatic problems in the tissue. The significantly higher number of BLIMP1^+^ cells suggests increased terminal differentiation of B lymphocytes to plasma cells (Shaffer et al., 2002; Turner et al., 1994). This goes in line with the elevated levels of plasma cells and antibodies including autoantibodies. Furthermore, high levels of CD95 receptor in spleens of knockout mice indicate enhanced CD95-dependent apoptotic activity, which could be associated with maturation of B cells (Hao et al., 2008; Rothstein et al., 1995). Several studies have shown that macrophages are important players in the induction of plasma cell terminal differentiation (Charo and Ransohoff, 2006; Sallusto and Baggiolini, 2008; Xu et al., 2012). In this study, we showed that the activation of B lymphocytes by macrophages significantly contributes to the development of autoantibodies. Indeed, MCPIP1-deficient macrophages triggered B-lymphocyte maturation, plasma cell expansion and autoantibody production *in vivo*. The plasma cell expansion can be linked to the fact that spleen tissue displayed significantly increased Baff expression, which activates B-cell responses. Moreover, IL-6, for which transcript is targeted by MCPIP1, was recently described as the essential growth and survival factor for plasmablasts (Jego et al., 2003; Matsushita et al., 2009; Mohr et al., 2009). Another upregulated cytokine, IL-5, was described to regulate genes involved in B-cell terminal maturation (Horikawa and Takatsu, 2006). Previous investigations failed to identify structural features that qualified IL-5 as an MCPIP1 substrate (Mino et al., 2015). However, the latest studies identified MCPIP1 as a regulator of the development and functions of IL-5-producing Th2 cells, which supports our findings (Peng et al., 2018). Furthermore, we observed a significant increase in *Cd40L* expression in knockout mice. CD40/CD40L interaction is required for the activation of humoral immune responses, including B-cell activation, plasma cell differentiation, antibody secretion and isotype switching (Malkiel et al., 2018; Uehata et al., 2013). SLE patients showed a significant increase in the CD40L-expressing monocytes (Katsiari et al., 2002), and recombinant CD40L induces the production of total IgG in B cells of SLE patients (Harigai et al., 1999), while CD40L overexpression triggers lupus-like autoimmune disease (Higuchi et al., 2002; Wang et al., 2003). Interestingly, only *Tnfrsf13b*/Taci, which is predominantly expressed on plasma cells and needed for the optimal generation of long-lived plasma cells, is upregulated in the spleen of knockout mice. Upregulation of transcript encoding BCMA indicates elevated plasma cell levels. BCMA is induced in late-memory B cells undergoing differentiation to antibody-producing B cells and is present on all plasma cells (Benson et al., 2008; Mackay et al., 2003; O'Connor et al., 2004). It seems to be important for the survival of long-lived plasma cells (O'Connor et al., 2004; Xu and Lam, 2001). Strongly downregulated Baff receptor/*Tnfrsf13c* that is present on mature B cells suggests that the mature B cells are a minor cell subpopulation and emphasize the disproportion in B-cell differentiation in knockout mice. During plasma cell differentiation, BCMA is induced, whereas Baff receptor is decreased (O'Connor et al., 2004). Exactly such an expression pattern was observed in MCPIP1 mutants compared to controls. However, the experiments with *Cd19*-cre-deleting strains crossed with mice carrying a floxed allele of *Mcpip1* are still needed to evaluate the role of MCPIP1 in B cells. B-cell-intrinsic sensitivity to relevant molecules such as Baff or Cd40L, and signs of autoimmunity-like production of autoantibodies and organ damage, need to be investigated. Nevertheless, these data emphasize the concept that macrophages orchestrate B-cell autoimmunity. Some activation pathways are intrinsic to B or T cells, but regulation processes may involve macrophages.

To our surprise, we observed low splenic T-cell count in the knockout mice, suggesting that healthy macrophage signaling supports not only B cells but also T-cell homeostasis. We detected very few signs of activation of the T cells in the *Mcpip1^fl/fl^-LysM^cre+^* mice, except for increased cytokine expression such as IFN-γ and a higher number of Th17 cells. Both IL-17 and IFN-γ are present in patients with SLE and correlate with disease severity (Bengtsson et al., 2000; Ma et al., 2010). The inconsistent low expression of *Rorc* could be a result of activation or differentiated status (Castro et al., 2017). Moreover, RORγt (*Rorc* gene product) is expressed not exclusively on Th17 cells but also in a subset of Tregs with enhanced immunosuppressive function (RORγt^+^ Tregs) (Ohnmacht et al., 2015; Sefik et al., 2015). We observed that there was a decrease in the percentage of other T-cell populations, including FoxP3^+^ Treg cells, in macrophage-specific MCPIP1-deficient mice. This was consistent with lower expression of Flt3L in the knockout mice, which facilitates the formation of Treg cells and reduces the severity of antigen-induced arthritis in mice (Svensson et al., 2013). Our data identify the control of autoimmunity as a novel function of MCPIP1. It controls *Tlr7*- and *Tlr9*-dependent activation of macrophages and therefore affects an important pathomechanism of SLE. MCPIP1-deficient macrophages exhibited enhanced production of proinflammatory cytokines, such as *Il5*, *Il6*, *Il17*, *Il22*, *Il23* and *Ifng*, but not type I IFN signaling. Whereas degradation of *Il6* mRNA has already been documented, the other transcripts have not been investigated experimentally (Liang et al., 2008; Matsushita et al., 2009; Mino et al., 2015; Wilamowski et al., 2018).

Overall, our results indicate that MCPIP1, which had previously unknown immunoregulatory effects in the context of spontaneous autoimmunity, is an important factor that limits the autoimmune renal phenotype. The severe autoinflammatory syndrome that develops in mice with a myeloid-specific MCPIP1 deficiency sheds light on the role of macrophages in processes of adaptive immunity.

## MATERIALS AND METHODS

### Animal studies

Myelomonocytic cell MCPIP1-deficient mice (*Mus musculus*) were generated and backcrossed to the C57BL/6 strain by crossing Lox-P-MCPIP1 mice with LysM-cre mice (The Jackson Laboratory). Female mice were housed in sterile filter-top cages with a 12 h dark/light cycle. All mice were sacrificed by cervical dislocation at 8 or 24 weeks of age. This study was carried out following the principles of the Directive 2010/63/EU on the Protection of Animals Used for Scientific Purpose and with approval by the local government authorities, II Lokalna Komisja Etyczna (local ethic commission) in Krakow.

### Evaluation of autoimmune tissue injury

Organs were fixed in 4% buffered formalin and embedded in paraffin. Histology staining was performed according to the manufacturer's instructions. We used the following antibodies: rat anti-BLIMP1 (cat. 14-5963-82, Invitrogen, 1:100), rabbit anti-CD95 (cat. bs-0215R-TR, Bioss, 1:400), rat anti-IRF4 (cat. sc-130921, Santa Cruz Biotechnology, 1:200), rat anti-B220 (cat. 14-0452-82, Invitrogen, 1:400), anti-CD3 (cat. MCA1477, Bio-Rad, 1:100), anti-F4/80 (cat. NB 600-404SS, NovusBio, 1:100) and anti-Ly6C (cat. MCA771GA, Serotec, 1:200). For quantitative analysis, glomerular cells were counted in ten cortical glomeruli per section (from at least eight to 16 mice in the group) or were analyzed using Adobe Photoshop CS4Extended (percentage of stained high-power field). Kidney function was determined by measuring serum BUN (DiaSys Diagnostic Systems), and serum creatinine levels were determined by the Jaffe method (DiaSys Diagnostic Systems). Albuminuria and proteinuria were determined via a mouse Albumin Quantification Set (Bethyl Laboratories, Montgomery, TX, USA) and the Bradford method, respectively.

### Flow cytometry

Anti-mouse CD3 FITC (cat. 553062, BD Biosciences), anti-CD4 APC (cat. 553051, BD Biosciences), anti-CD8a PerCP (cat. 553030, BD Biosciences), anti-CD25 PB (cat. 553072, BD Biosciences), anti-FoxP3 (BioLegend, San Diego, USA), anti-CD11c PE (cat. 557401, BD Biosciences), anti-F4/80 APC (AbDSeroTec, Düsseldorf, Germany), anti-CD86 FITC (cat. 553691, BD Biosciences), BV421 rat anti-mouse CD18 Clone C71/16 (RUO), anti-CD19 PB (cat. 115526, BioLegend), anti-MHC II (I-A/I-E) (cat. 107605, BioLegend), B220 Alexa Fluor 647 (cat. 103229, BioLegend), anti-mouse kappa light chain PE (cat. 559940, BD Biosciences), anti-CD138 APC (cat. 558626, BD Biosciences), anti-CD11b PECy7 (cat. 101216, BioLegend), anti-CD11c FITC (cat. 117306, BioLegend), anti-MHCII PerCP Cy5.5 (cat. 107626, BioLegend), anti-CD16/32 (cat. 101302, BioLegend), anti-cd80 violett650 (cat. 104732, BioLegend), anti-CD317 PE (cat. 127104, BioLegend) and mouse anti-Th17/Tregs Phenotyping Kit (cat. 560767, BD Biosciences) were used to differentiate T- and B-cell subsets as well as dendritic cells and macrophages. Intracellular labeling (FoxP3 and kappa light chain) was done using a Cytofix/Cytoperm Kit (BD Biosciences), following the manufacturer's instructions. Plasma cells were analyzed after doublet and dead cell exclusion (Zombie viability dye) from lymphocytes (scatter gate) gating for MHCII^+^CD138^+^ and kappa light chain-positive cells. Staining for the kappa light chain was done using BD Biosciences intracellular staining, following the manufacturer's instructions. Cell-counting beads (Invitrogen) were used for determining cell numbers by FACS Cytoflex (Beckman Coulter, Indianapolis, IN, USA) and analyzed with FlowJo V.10 Software (BD Biosciences).

### Quantitative real-time PCR

SYBR Green Dye detection system was used for quantitative real-time PCR on a Light Cycler 480 (Roche, Mannheim, Germany). Gene-specific primers (225 nM; Metabion, Martinsried, Germany) were used as listed in Table S1. Standard controls for genomic DNA contamination, RNA quality and general PCR performance were included.

### Autoantibody and plasma cytokine analysis

For detection of antibodies to dsDNA, NUNC MaxiSorp ELISA plates (Thermo Fisher Scientific) were coated with poly-L-lysine (Trevigen) and PBS (ratio 1:1) for 1 h. Plates were washed with TrisNaCl (50 mM Tris and 0.14 M NaCl pH 7.5) and were coated with dsDNA (2 µg/ml) in saline-sodium citrate (SSC) pH 7.0 buffer overnight. For anti-nucleosome antibodies, MaxiSorp enzyme-linked immunosorbent assay (ELISA) plates were pre-coated with poly-L-lysine and PBS (1:1) for 1 h. Plates were washed with TrisNaCl buffer and incubated with mouse DNA histones (2 µg/ml) in SSC buffer overnight at 4°C. For anti-Smith antibodies, MaxiSorp ELISA plates were coated with Smith antigen (Sm3000; Immunovision) in 0.05 M carbonate-bicarbonate buffer overnight at 4°C. Serum samples were diluted 1:100 to 1:1,000,000 for all IgM/IgG ELISAs. Sera from 24week-old C57BL/6*^lpr/lpr^* and 24-week-old MRL*^lpr/lpr^* mice, as well as IgG-poor serum, were used as controls. Horseradish peroxidase-conjugated antibody to mouse IgG and IgM (A90-131P and A90-101A, Bethyl) was used as a secondary antibody (1:50,000). Absorbance was measured at 450 nm with a Sun-rise plate reader (TECAN).

### *In vitro* experiments

Bone marrow cells from wild-type and knockout mice were isolated from femur and tibia, erythrocyte depleted and cultured with 20 ng/ml mouse recombinant M-CSF (Immunotools, Germany) in Dulbecco's modified Eagle medium (DMEM) supplemented with 10% fetal calf serum (FCS) and 1% penicillin/streptomycin for 7 days to generate macrophages. On day 7, cells were stimulated with 10 ng/ml LPS (Sigma-Aldrich, USA), 0.3 µM CpG ODN 2006 (Hycult Biotechnology, The Netherlands), 100 ng/ml R848 (Enzo Life Science, NY, USA) or left untreated as a control. Splenocyte cells from wild-type and knockout mice were isolated from fresh spleens, erythrocyte depleted and cultured in DMEM supplemented with 10% FCS and 1% penicillin/streptomycin for 24 h. The next day, cells were stimulated with 10 ng/ml IFN-γ, 10 ng/ml IL-6 or left untreated as a control. All ligands and cytokines were obtained from Invivogen and Immunotools, respectively. After 4 h and 24 h, macrophages and splenocytes were processed for further analysis as indicated in the Results section.

The MACS kit purchased from Miltenyi Biotec was used for purification of the B-cell subset from spleen cell suspension of wild-type and knockout mice. Isolated cells were seeded for 48 h stimulation with 1%, 20% and 50% wild-type and knockout murine serum on 96-well plates. Proliferation of the cells was detected with the thymidine analog 5-bromo-2′-deoxyuridine (BrdU) following its incorporation into newly synthesized DNA and its subsequent detection with an anti-BrdU antibody (Roche), following manual instruction. Absorbance was measured at 450 nm using FlexStation (Molecular Devices). The A20 B-lymphocyte cell line (ATCC TIB-208) was exposed on 96-well plates to 20% wild-type and knockout murine serum and 0.3 µM CpG ODN 2006 for 48 h. MTT assay was performed according to a commercial manual; absorbance was measured at 570 nm.

### Statistical analysis

Data were expressed as mean±s.e.m. Data from wild-type and knockout mice were compared with ANOVA on ranks, followed by the Student–Newman-Keuls test using SigmaStat Software (Jandel Scientific, Erkrath, Germany) (Figs. 2D, 5B, 6B). Student’s *t*-test was used for direct comparisons between single groups, i.e. wild-type and knockout cells/mice in cases of normally distributed data or sample size *n*>15 (Figs. 1I, 2A, 3B, 5A,D-F). Mann–Whitney U test was used to analyze data with a small sample size and non-parametric distribution (all other figures). We used GraphPad Prism software for the analyses. *P*<0.05 indicated statistical significance.

## Supplementary Material

Supplementary information

## References

[DMM047589C1] Bengtsson, A. A., Sturfelt, G., Truedsson, L., Blomberg, J., Alm, G., Vallin, H. and Rönnblom, L. (2000). Activation of type I interferon system in systemic lupus erythematosus correlates with disease activity but not with antiretroviral antibodies. *Lupus* 9, 664-671. 10.1191/096120300674499064 11199920

[DMM047589C2] Benson, M. J., Dillon, S. R., Castigli, E., Geha, R. S., Xu, S., Lam, K.-P. and Noelle, R. J. (2008). Cutting edge: the dependence of plasma cells and independence of memory B cells on BAFF and APRIL. *J. Immunol.* 180, 3655-3659. 10.4049/jimmunol.180.6.3655 18322170

[DMM047589C3] Birnberg, T., Bar-On, L., Sapoznikov, A., Caton, M. L., Cervantes-Barragán, L., Makia, D., Krauthgamer, R., Brenner, O., Ludewig, B., Brockschnieder, D. et al. (2008). Lack of conventional dendritic cells is compatible with normal development and T Cell homeostasis, but causes myeloid proliferative syndrome. *Immunity* 29, 986-997. 10.1016/j.immuni.2008.10.012 19062318

[DMM047589C4] Castro, G., Liu, X., Ngo, K., De Leon-Tabaldo, A., Zhao, S., Luna-Roman, R., Yu, J., Cao, T., Kuhn, R., Wilkinson, P. et al. (2017). RORγt and RORα signature genes in human Th17 cells. *PLoS ONE* 12, e0181868. 10.1371/journal.pone.0181868 28763457 PMC5538713

[DMM047589C5] Charo, I. F. and Ransohoff, R. M. (2006). The many roles of chemokines and chemokine receptors in inflammation. *N. Engl. J. Med.* 354, 610-621. 10.1056/NEJMra052723 16467548

[DMM047589C6] Clausen, B. E., Burkhardt, C., Reith, W., Renkawitz, R. and Förster, I. (1999). Conditional gene targeting in macrophages and granulocytes using LysMcre mice. *Transgenic Res.* 8, 265-277. 10.1023/A:1008942828960 10621974

[DMM047589C7] Dobosz, E., Wilamowski, M., Lech, M., Bugara, B., Jura, J., Potempa, J. and Koziel, J. (2016). MCPIP-1, Alias Regnase-1, controls epithelial inflammation by posttranscriptional regulation of IL-8 production. *J. Innate Immun.* 8, 564-578. 10.1159/000448038 27513529 PMC5089914

[DMM047589C8] Fond, A. M. and Ravichandran, K. S. (2016). Clearance of dying cells by phagocytes: mechanisms and implications for disease pathogenesis. *Adv. Exp. Med. Biol.* 930, 25-49. 10.1007/978-3-319-39406-0_2 27558816 PMC6721615

[DMM047589C9] Garg, A. V., Amatya, N., Chen, K., Cruz, J. A., Grover, P., Whibley, N., Conti, H. R., Hernandez Mir, G., Sirakova, T., Childs, E. C. et al. (2015). MCPIP1 endoribonuclease activity negatively regulates interleukin-17-mediated signaling and inflammation. *Immunity* 43, 475-487. 10.1016/j.immuni.2015.07.021 26320658 PMC4575280

[DMM047589C10] Hao, Z., Duncan, G. S., Seagal, J., Su, Y.-W., Hong, C., Haight, J., Chen, N.-J., Elia, A., Wakeham, A., Li, W. Y. et al. (2008). Fas receptor expression in germinal-center B cells is essential for T and B lymphocyte homeostasis. *Immunity* 29, 615-627. 10.1016/j.immuni.2008.07.016 18835195 PMC3470429

[DMM047589C11] Harigai, M., Hara, M., Fukasawa, C., Nakazawa, S., Kawaguchi, Y., Kamatani, N. and Kashiwazaki, S. (1999). Responsiveness of peripheral blood B cells to recombinant CD40 ligand in patients with systemic lupus erythematosus. *Lupus* 8, 227-233. 10.1191/096120399678847678 10342716

[DMM047589C12] Higuchi, T., Aiba, Y., Nomura, T., Matsuda, J., Mochida, K., Suzuki, M., Kikutani, H., Honjo, T., Nishioka, K. and Tsubata, T. (2002). Cutting edge: ectopic expression of CD40 ligand on B cells induces lupus-like autoimmune disease. *J. Immunol.* 168, 9-12. 10.4049/jimmunol.168.1.9 11751940

[DMM047589C13] Horikawa, K. and Takatsu, K. (2006). Interleukin-5 regulates genes involved in B-cell terminal maturation. *Immunology* 118, 497-508. 10.1111/j.1365-2567.2006.02382.x 16895557 PMC1782313

[DMM047589C14] Jain, A. and Pasare, C. (2017). Innate control of adaptive immunity: beyond the three-signal paradigm. *J. Immunol.* 198, 3791-3800. 10.4049/jimmunol.1602000 28483987 PMC5442885

[DMM047589C15] Jego, G., Palucka, A. K., Blanck, J.-P., Chalouni, C., Pascual, V. and Banchereau, J. (2003). Plasmacytoid dendritic cells induce plasma cell differentiation through type I interferon and interleukin 6. *Immunity* 19, 225-234. 10.1016/S1074-7613(03)00208-5 12932356

[DMM047589C16] Jonuleit, H., Schmitt, E., Schuler, G., Knop, J. and Enk, A. H. (2000). Induction of interleukin 10–producing, nonproliferating CD4+ T cells with regulatory properties by repetitive stimulation with allogeneic immature human dendritic cells. *J. Exp. Med.* 192, 1213-1222. 10.1084/jem.192.9.1213 11067871 PMC2193357

[DMM047589C17] Jura, J., Skalniak, L. and Koj, A. (2012). Monocyte chemotactic protein-1-induced protein-1 (MCPIP1) is a novel multifunctional modulator of inflammatory reactions. *Biochim. Biophys. Acta (BBA) Mol. Cell Res.* 1823, 1905-1913. 10.1016/j.bbamcr.2012.06.029 22771441

[DMM047589C18] Katsiari, C. G., Liossis, S.-N. C., Souliotis, V. L., Dimopoulos, A. M., Manoussakis, M. N. and Sfikakis, P. P. (2002). Aberrant expression of the costimulatory molecule CD40 ligand on monocytes from patients with systemic lupus erythematosus. *Clin. Immunol.* 103, 54-62. 10.1006/clim.2001.5172 11987985

[DMM047589C19] Lamagna, C., Scapini, P., Van Ziffle, J. A., DeFranco, A. L. and Lowell, C. A. (2013). Hyperactivated MyD88 signaling in dendritic cells, through specific deletion of Lyn kinase, causes severe autoimmunity and inflammation. *Proc. Natl. Acad. Sci. USA* 110, E3311-E3320. 10.1073/pnas.1300617110 23940344 PMC3761623

[DMM047589C20] Lech, M. and Anders, H.-J. (2013). The pathogenesis of lupus nephritis. *J. Am. Soc. Nephrol.* 24, 1357-1366. 10.1681/ASN.2013010026 23929771 PMC3752952

[DMM047589C21] Li, Y., Huang, X., Huang, S., He, H., Lei, T., Saaoud, F., Yu, X.-Q., Melnick, A., Kumar, A., Papasian, C. J. et al. (2017). Central role of myeloid MCPIP1 in protecting against LPS-induced inflammation and lung injury. *Signal Transduct. Target. Ther.* 2, 17066. 10.1038/sigtrans.2017.66 29263935 PMC5721545

[DMM047589C22] Liang, J., Wang, J., Azfer, A., Song, W., Tromp, G., Kolattukudy, P. E. and Fu, M. (2008). A novel CCCH-zinc finger protein family regulates proinflammatory activation of macrophages. *J. Biol. Chem.* 283, 6337-6346. 10.1074/jbc.M707861200 18178554

[DMM047589C23] Liang, J., Saad, Y., Lei, T., Wang, J., Qi, D., Yang, Q., Kolattukudy, P. E. and Fu, M. (2010). MCP-induced protein 1 deubiquitinates TRAF proteins and negatively regulates JNK and NF-κB signaling. *J. Exp. Med.* 207, 2959-2973. 10.1084/jem.20092641 21115689 PMC3005225

[DMM047589C24] Liu, H., Fang, S., Wang, W., Cheng, Y., Zhang, Y., Liao, H., Yao, H. and Chao, J. (2016). Macrophage-derived MCPIP1 mediates silica-induced pulmonary fibrosis via autophagy. *Part. Fibre Toxicol.* 13, 55. 10.1186/s12989-016-0167-z 27782836 PMC5078901

[DMM047589C25] Ma, S., Pathak, S., Trinh, L. and Lu, R. (2008). Interferon regulatory factors 4 and 8 induce the expression of Ikaros and Aiolos to down-regulate pre-B-cell receptor and promote cell-cycle withdrawal in pre-B-cell development. *Blood* 111, 1396-1403. 10.1182/blood-2007-08-110106 17971486 PMC2214771

[DMM047589C26] Ma, J., Yu, J., Tao, X., Cai, L., Wang, J. and Zheng, S. G. (2010). The imbalance between regulatory and IL-17-secreting CD4+ T cells in lupus patients. *Clin. Rheumatol.* 29, 1251-1258. 10.1007/s10067-010-1510-7 20563617

[DMM047589C27] Mackay, F., Schneider, P., Rennert, P. and Browning, J. (2003). BAFF AND APRIL: a tutorial on B cell survival. *Annu. Rev. Immunol.* 21, 231-264. 10.1146/annurev.immunol.21.120601.141152 12427767

[DMM047589C28] Malkiel, S., Barlev, A. N., Atisha-Fregoso, Y., Suurmond, J. and Diamond, B. (2018). Plasma cell differentiation pathways in systemic lupus erythematosus. *Front. Immunol.* 9, 427. 10.3389/fimmu.2018.00427 29556239 PMC5845388

[DMM047589C29] Matsushita, K., Takeuchi, O., Standley, D. M., Kumagai, Y., Kawagoe, T., Miyake, T., Satoh, T., Kato, H., Tsujimura, T., Nakamura, H. et al. (2009). Zc3h12a is an RNase essential for controlling immune responses by regulating mRNA decay. *Nature* 458, 1185-1190. 10.1038/nature07924 19322177

[DMM047589C30] Mencarelli, A., Khameneh, H. J., Fric, J., Vacca, M., El Daker, S., Janela, B., Tang, J. P., Nabti, S., Balachander, A., Lim, T. S. et al. (2018). Calcineurin-mediated IL-2 production by CD11chighMHCII+ myeloid cells is crucial for intestinal immune homeostasis. *Nat. Commun.* 9, 1102. 10.1038/s41467-018-03495-3 29549257 PMC5856784

[DMM047589C31] Merayo-Chalico, J., Rajme-López, S., Barrera-Vargas, A., Alcocer-Varela, J., Díaz-Zamudio, M. and Gómez-Martín, D. (2016). Lymphopenia and autoimmunity: a double-edged sword. *Hum. Immunol.* 77, 921-929. 10.1016/j.humimm.2016.06.016 27343993

[DMM047589C32] Miao, R., Huang, S., Zhou, Z., Quinn, T., Van Treeck, B., Nayyar, T., Dim, D., Jiang, Z., Papasian, C. J., Eugene Chen, Y. et al. (2013). Targeted disruption of MCPIP1/Zc3h12a results in fatal inflammatory disease. *Immunol. Cell Biol.* 91, 368-376. 10.1038/icb.2013.11 23567898 PMC3932977

[DMM047589C33] Mino, T., Murakawa, Y., Fukao, A., Vandenbon, A., Wessels, H.-H., Ori, D., Uehata, T., Tartey, S., Akira, S., Suzuki, Y. et al. (2015). Regnase-1 and roquin regulate a common element in inflammatory mRNAs by spatiotemporally distinct mechanisms. *Cell* 161, 1058-1073. 10.1016/j.cell.2015.04.029 26000482

[DMM047589C34] Mistry, P. and Kaplan, M. J. (2017). Cell death in the pathogenesis of systemic lupus erythematosus and lupus nephritis. *Clin. Immunol.* 185, 59-73. 10.1016/j.clim.2016.08.010 27519955 PMC5299061

[DMM047589C35] Mizgalska, D., Węgrzyn, P., Murzyn, K., Kasza, A., Koj, A., Jura, J., Jarząb, B. and Jura, J. (2009). Interleukin-1-inducible MCPIP protein has structural and functional properties of RNase and participates in degradation of IL-1β mRNA. *FEBS J.* 276, 7386-7399. 10.1111/j.1742-4658.2009.07452.x 19909337

[DMM047589C36] Mohr, E., Serre, K., Manz, R. A., Cunningham, A. F., Khan, M., Hardie, D. L., Bird, R. and MacLennan, I. C. M. (2009). Dendritic cells and Monocyte/Macrophages that create the IL-6/APRIL-rich lymph node microenvironments where plasmablasts mature. *J. Immunol.* 182, 2113-2123. 10.4049/jimmunol.0802771 19201864

[DMM047589C100] Mosser, D. M., Hamidzadeh, K. and Goncalves, R. (2021). Macrophages and the maintenance of homeostasis. *Cell Mol. Immunol.* 18, 579-587. 10.1038/s41423-020-00541-3 32934339 PMC7491045

[DMM047589C37] Navegantes, K. C., de Souza Gomes, R., Pereira, P. A. T., Czaikoski, P. G., Azevedo, C. H. M. and Monteiro, M. C. (2017). Immune modulation of some autoimmune diseases: the critical role of macrophages and neutrophils in the innate and adaptive immunity. *J. Transl. Med.* 15, 36. 10.1186/s12967-017-1141-8 28202039 PMC5312441

[DMM047589C38] O'Connor, B. P., Raman, V. S., Erickson, L. D., Cook, W. J., Weaver, L. K., Ahonen, C., Lin, L.-L., Mantchev, G. T., Bram, R. J. and Noelle, R. J. (2004). BCMA is essential for the survival of long-lived bone marrow plasma cells. *J. Exp. Med.* 199, 91-98. 10.1084/jem.20031330 14707116 PMC1887725

[DMM047589C39] Ohnmacht, C., Pullner, A., King, S. B. S., Drexler, I., Meier, S., Brocker, T. and Voehringer, D. (2009). Constitutive ablation of dendritic cells breaks self-tolerance of CD4 T cells and results in spontaneous fatal autoimmunity. *J. Exp. Med.* 206, 549-559. 10.1084/jem.20082394 19237601 PMC2699126

[DMM047589C40] Ohnmacht, C., Park, J.-H., Cording, S., Wing, J. B., Atarashi, K., Obata, Y., Gaboriau-Routhiau, V., Marques, R., Dulauroy, S., Fedoseeva, M. et al. (2015). The microbiota regulates type 2 immunity through RORγt+ T cells. *Science* 349, 989-993. 10.1126/science.aac4263 26160380

[DMM047589C41] Pan, Q., Chen, J., Guo, L., Lu, X., Liao, S., Zhao, C., Wang, S. and Liu, H. (2019). Mechanistic insights into environmental and genetic risk factors for systemic lupus erythematosus. *Am. J. Transl. Res.* 11, 1241-1254. 30972159 PMC6456562

[DMM047589C42] Peng, H., Ning, H., Wang, Q., Lu, W., Chang, Y., Wang, T. T., Lai, J., Kolattukudy, P. E., Hou, R., Hoft, D. F. et al. (2018). Monocyte chemotactic protein–induced protein 1 controls allergic airway inflammation by suppressing IL-5–producing TH2 cells through the Notch/Gata3 pathway. *J. Allergy Clin. Immunol.* 142, 582-594.e10. 10.1016/j.jaci.2017.09.031 29111212 PMC5924426

[DMM047589C43] Petersone, L., Edner, N. M., Ovcinnikovs, V., Heuts, F., Ross, E. M., Ntavli, E., Wang, C. J. and Walker, L. S. K. (2018). T Cell/B cell collaboration and autoimmunity: an intimate relationship. *Front. Immunol.* 9, 1941. 10.3389/fimmu.2018.01941 30210496 PMC6119692

[DMM047589C44] Raker, V. K., Domogalla, M. P. and Steinbrink, K. (2015). Tolerogenic dendritic cells for regulatory T cell induction in man. *Front. Immunol.* 6, 569. 10.3389/fimmu.2015.00569 26617604 PMC4638142

[DMM047589C45] Raymond, W., Ostli Eilertsen, G., Griffiths, S. and Nossent, J. (2017). IL-17A levels in systemic lupus erythematosus associated with inflammatory markers and lower rates of malignancy and heart damage: Evidence for a dual role. *Eur. J. Rheumatol.* 4, 29-35. 10.5152/eurjrheum.2017.16059 28293450 PMC5335884

[DMM047589C46] Rothstein, T. L., Wang, J. K. M., Panka, D. J., Foote, L. C., Wang, Z., Stanger, B., Cui, H., Ju, S.-T. and Marshak-Rothstein, A. (1995). Protection against Fas-dependent Thl-mediated apoptosis by antigen receptor engagement in B cells. *Nature* 374, 163-165. 10.1038/374163a0 7533263

[DMM047589C47] Sallusto, F. and Baggiolini, M. (2008). Chemokines and leukocyte traffic. *Nat. Immunol.* 9, 949-952. 10.1038/ni.f.214 18711431

[DMM047589C48] Sato, K., Yamashita, N., Baba, M. and Matsuyama, T. (2003). Modified myeloid dendritic cells act as regulatory dendritic cells to induce anergic and regulatory T cells. *Blood* 101, 3581-3589. 10.1182/blood-2002-09-2712 12511411

[DMM047589C49] Sciammas, R., Shaffer, A. L., Schatz, J. H., Zhao, H., Staudt, L. M. and Singh, H. (2006). Graded expression of interferon regulatory factor-4 coordinates isotype switching with plasma cell differentiation. *Immunity* 25, 225-236. 10.1016/j.immuni.2006.07.009 16919487

[DMM047589C50] Sefik, E., Geva-Zatorsky, N., Oh, S., Konnikova, L., Zemmour, D., McGuire, A. M., Burzyn, D., Ortiz-Lopez, A., Lobera, M., Yang, J. et al. (2015). Individual intestinal symbionts induce a distinct population of RORγ+ regulatory T cells. *Science* 349, 993-997. 10.1126/science.aaa9420 26272906 PMC4700932

[DMM047589C51] Shaffer, A. L., Lin, K.-I., Kuo, T. C., Yu, X., Hurt, E. M., Rosenwald, A., Giltnane, J. M., Yang, L., Zhao, H., Calame, K. et al. (2002). Blimp-1 orchestrates plasma cell differentiation by extinguishing the mature B cell gene expression program. *Immunity* 17, 51-62. 10.1016/S1074-7613(02)00335-7 12150891

[DMM047589C52] Simonetti, G., Carette, A., Silva, K., Wang, H., De Silva, N. S., Heise, N., Siebel, C. W., Shlomchik, M. J. and Klein, U. (2013). IRF4 controls the positioning of mature B cells in the lymphoid microenvironments by regulating NOTCH2 expression and activity. *J. Exp. Med.* 210, 2887-2902. 10.1084/jem.20131026 24323359 PMC3865479

[DMM047589C53] Skalniak, L., Mizgalska, D., Zarebski, A., Wyrzykowska, P., Koj, A. and Jura, J. (2009). Regulatory feedback loop between NF-κB and MCP-1-induced protein 1 RNase. *FEBS J.* 276, 5892-5905. 10.1111/j.1742-4658.2009.07273.x 19747262

[DMM047589C54] Svensson, M. N. D., Andersson, S. E. M., Erlandsson, M. C., Jonsson, I.-M., Ekwall, A.-K. H., Andersson, K. M. E., Nilsson, A., Bian, L., Brisslert, M. and Bokarewa, M. I. (2013). Fms-like tyrosine kinase 3 ligand controls formation of regulatory T cells in autoimmune arthritis. *PLoS ONE* 8, e54884. 10.1371/journal.pone.0054884 23349985 PMC3549988

[DMM047589C55] Theofilopoulos, A. N., Dummer, W. and Kono, D. H. (2001). T cell homeostasis and systemic autoimmunity. *J. Clin. Investig.* 108, 335-340. 10.1172/JCI200112173 11489923 PMC209358

[DMM047589C56] Thomas, J. W. (2001). Antigen-specific responses in autoimmunity and tolerance. *Immunol. Res.* 23, 235-244. 10.1385/IR:23:2-3:235 11444388

[DMM047589C57] Turner, C. A., Mack, D. H. and Davis, M. M. (1994). Blimp-1, a novel zinc finger-containing protein that can drive the maturation of B lymphocytes into immunoglobulin-secreting cells. *Cell* 77, 297-306. 10.1016/0092-8674(94)90321-2 8168136

[DMM047589C58] Uehata, T., Iwasaki, H., Vandenbon, A., Matsushita, K., Hernandez-Cuellar, E., Kuniyoshi, K., Satoh, T., Mino, T., Suzuki, Y., Standley, D. M. et al. (2013). Malt1-induced cleavage of regnase-1 in CD4^+^ helper T cells regulates immune activation. *Cell* 153, 1036-1049. 10.1016/j.cell.2013.04.034 23706741

[DMM047589C59] Wang, X., Huang, W., Schiffer, L. E., Mihara, M., Akkerman, A., Hiromatsu, K. and Davidson, A. (2003). Effects of anti-CD154 treatment on B cells in murine systemic lupus erythematosus. *Arthritis. Rheum.* 48, 495-506. 10.1002/art.10929 12571860

[DMM047589C60] Wilamowski, M., Gorecki, A., Dziedzicka-Wasylewska, M. and Jura, J. (2018). Substrate specificity of human MCPIP1 endoribonuclease. *Sci. Rep.* 8, 7381. 10.1038/s41598-018-25765-2 29743536 PMC5943514

[DMM047589C61] Xu, S. and Lam, K.-P. (2001). B-cell maturation protein, which binds the tumor necrosis factor family members BAFF and APRIL, is dispensable for humoral immune responses. *Mol. Cell. Biol.* 21, 4067-4074. 10.1128/MCB.21.12.4067-4074.2001 11359913 PMC87068

[DMM047589C62] Xu, W., Joo, H. M., Clayton, S., Dullaers, M., Herve, M.-C., Blankenship, D., De La Morena, M. T., Balderas, R., Picard, C., Casanova, J.-L. et al. (2012). Macrophages induce differentiation of plasma cells through CXCL10/IP-10. *J. Exp. Med.* 209, 1813-1823. 10.1084/jem.20112142 22987802 PMC3457728

[DMM047589C63] Zhou, L., Azfer, A., Niu, J., Graham, S., Choudhury, M., Adamski, F. M., Younce, C., Binkley, P. F. and Kolattukudy, P. E. (2006). Monocyte chemoattractant protein-1 induces a novel transcription factor that causes cardiac myocyte apoptosis and ventricular dysfunction. *Circ. Res.* 98, 1177-1185. 10.1161/01.RES.0000220106.64661.71 16574901 PMC1523425

