# Supplementary material for: Murine myeloid cell MCPIP1 suppresses autoimmunity by regulating B-cell expansion and differentiation
Source: Dis Model Mech. 2021 Mar 18;14(3):dmm047589. doi: 10.1242/dmm.047589 (PMC7988765; doi:10.1242/dmm.047589)
Supplement: Supplementary information [file dmm-14-047589-s1.pdf]

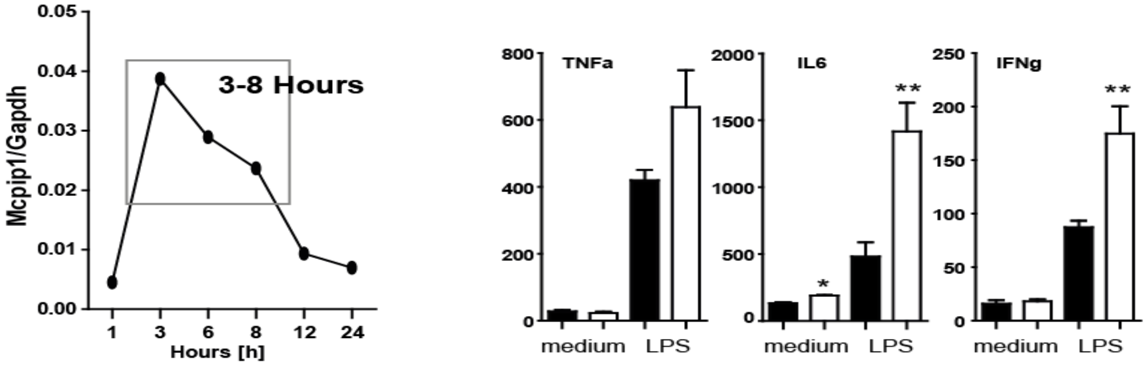

Fig. S1. Bone marrow-derived macrophages and LPS stimulation A: bone marrow derived macrophages were stimulated with 2 ng/ml ultrapure LPS for 1 to 24 hours as indicated in the figure. mRNA expression of Mcpip1 was measured by RT-qPCR. B: knockout and control macrophages were stimulated with LPS for 6 hours levels of TNF-α, IL-6 and IFN-γ were measured by ELISA. Data represent means ± SEM from 3 independent stimulations. ** p<0.01.

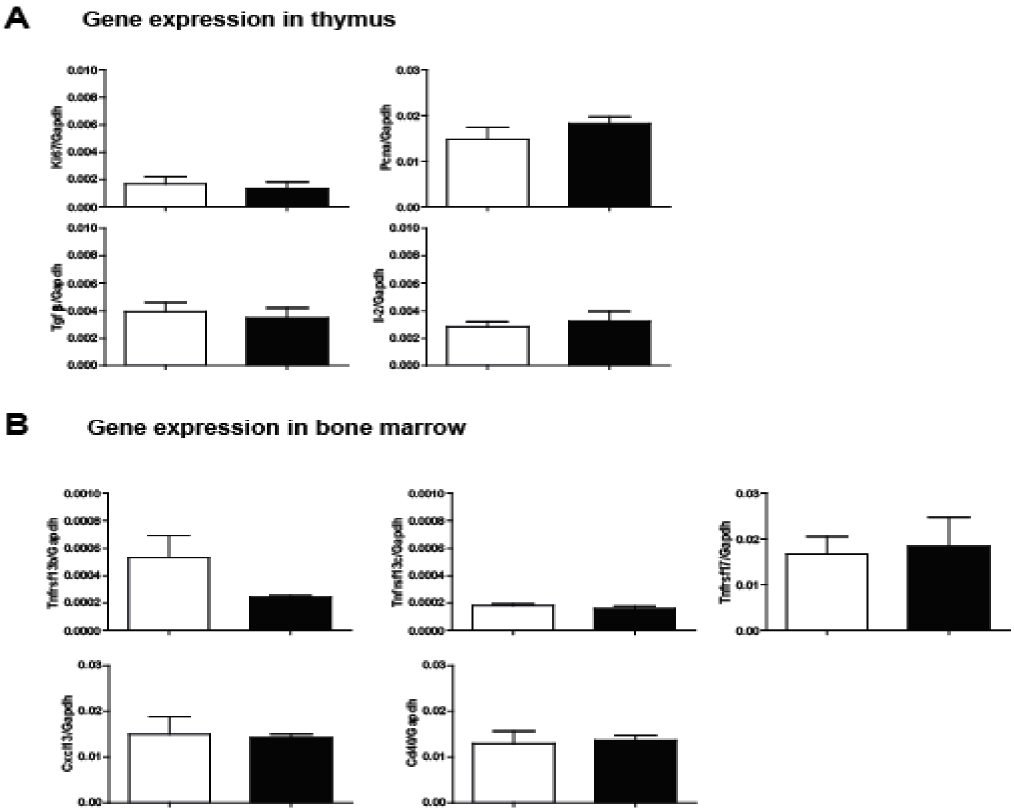

Fig. S2. Gene expression in thymus and bone marrow. RNA was isolated from knockout and control mice for real-time PCR analysis. Data are means ± SEM from at least 8 mice per group, versus control mice.

Disease Models & Mechanisms • Supplementary information

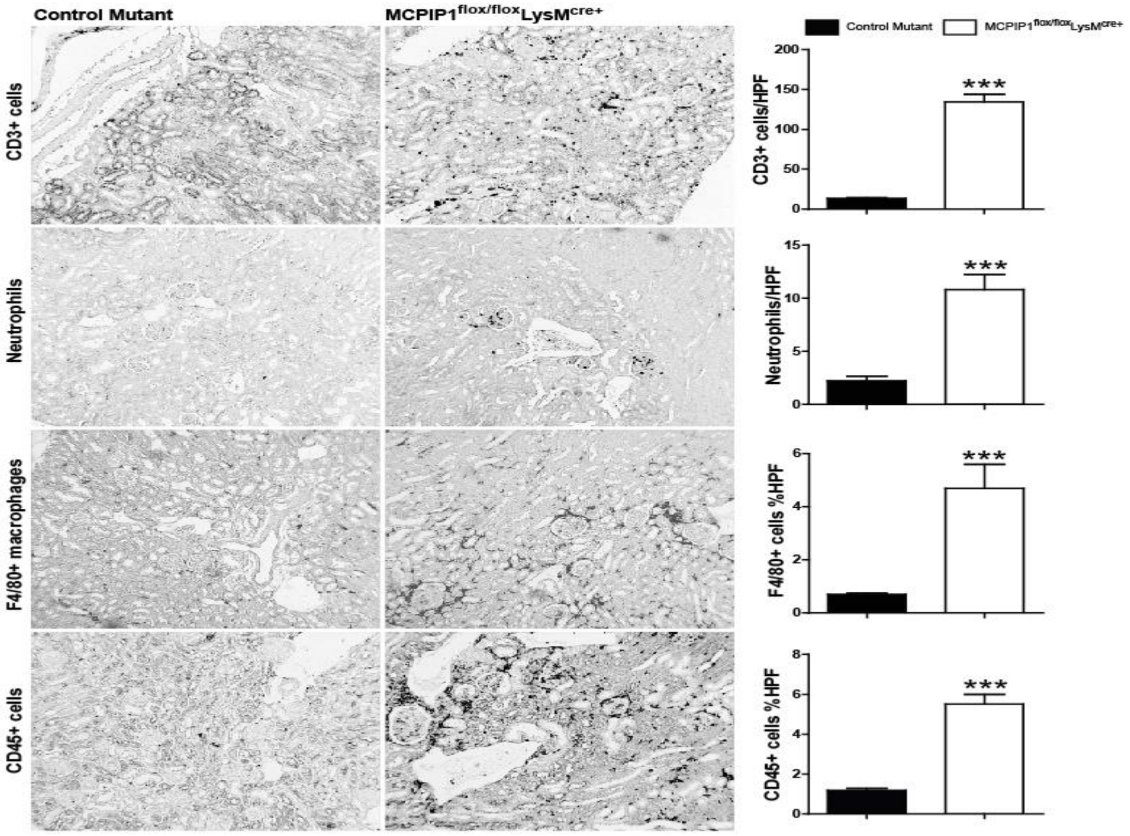

Fig. S3. Renal immune cell infiltration. Kidney sections were stained with CD3+, CD45+, F4/80+, and Neutrophils and quantified by counting (CD3, Neutrophils) or by using a semi-quantitative score (CD45, F4/80). Data are shown as means of at least 40 scored HPF or quantified in glomeruli from at least 8 mice in each group; *** p<0.001.

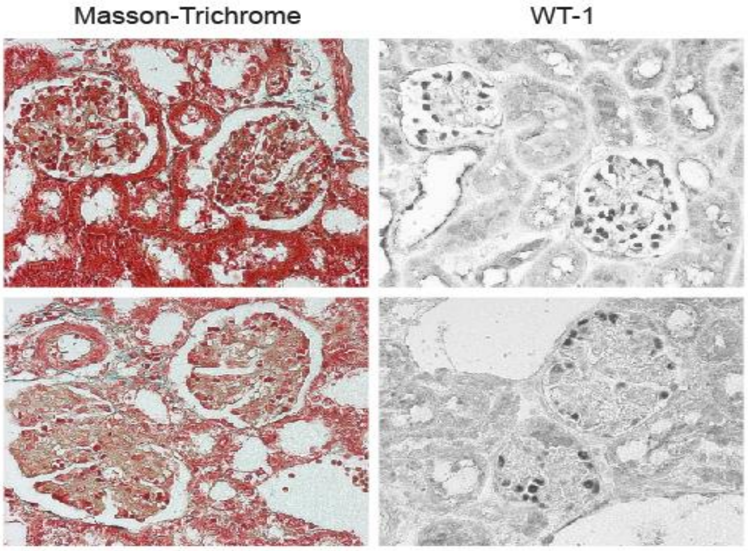

Fig. S4. Renal fibrosis and podocyte staining. Kidney sections were stained Masson Trichrome and WT-1 (podocyte marker) to visualize fibrosis and podocyte loss respectively.

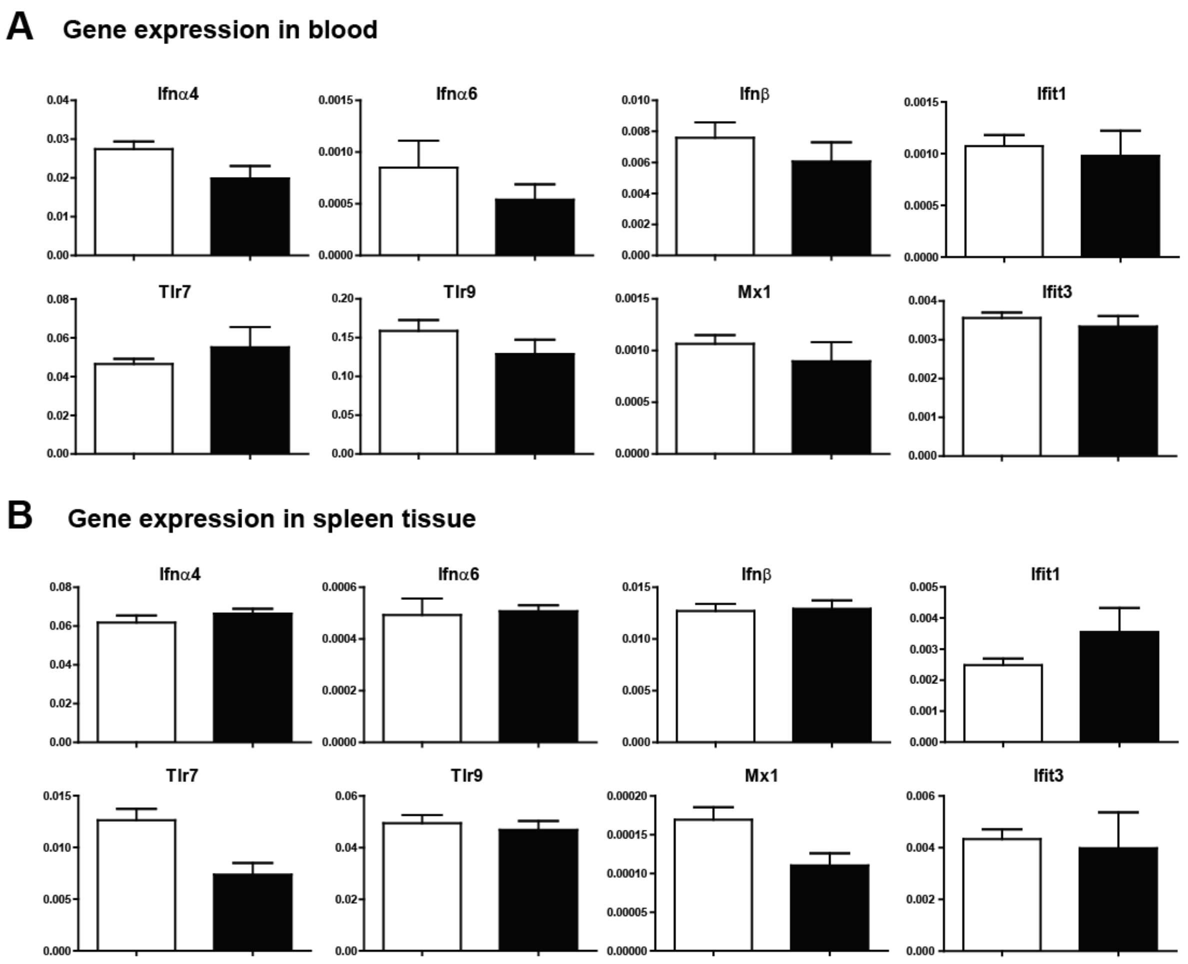

Fig. S5. Gene expression (relative to Gapdh) in whole blood (A) and spleen (B). RNA was isolated from knockout and control mice for real-time PCR analysis. Data are means ± SEM from at least 8 mice per group, versus control mice.

Disease Models & Mechanisms • Supplementary information

Table S1. Primers used for RT-PCR

Gene name	Accession Nr.	Sequence Forward Primer	Sequence Reverse Proimer.
Bcl 6	NM_009744	.: 5'-AGTTTCTAGGAAAGGCCGGA-3'	.: 5'-ACTAGCGTGCCGGGTAAACT-3'
Bcl 10	NM_009740	.: 5'-ACGGAGGAGGATTTGACTGA-3'	.: 5'-TGCACGTAGATGATCAAAATGTC-3'
Ccl 28	NM_020279	.: 5'-GTTCATGCAGCATCCAGAGAGC-3'	.: 5'-TCTGAGGCTCTCATCCACTGCT-3'
Cd 40	NM_011611	.: 5'-ACCAGCAAGGATTGCGAGGCAT-3'	.: 5'-GGATGACAGACGGTATCAGTGG-3'
Cd40l	NM_011616	.: 5'-GAACTGTGAGGAGATGAGAAGGC-3'	.: 5'-TGGCTTCGCTTACAACGTGTGC-3'
Cxcl 13	NM_018866	.: 5'-CATAGATCGGATTCAAGTTACGCC-3'	.: 5'-GTAACCATTTGGCACGAGGATTC-3'
Flt3l	NM_010228	.: 5'-TGGATGAGCAGTGTGAACCGCT-3'	.: 5'-GCCAAATGCAGAGGCTTGAACG-3'
Foxp3	NM_001199347	.: 5'-TTCATGCATCAGCTCTCCAC-3'	.: 5'-CTGGACACCCATTCCAGACT-3'
Gapdh	NM_001289726	.: 5'-CATGGCCTTCCGTGTTCCTA-3'	.: 5'-CCTGCTTCACCACCTTCTCA-3'
Gata3	NM_001355110	.: 5'-GCCTGCGGACTCTACCATAA-3'	.: 5'-AGGATGTCCCTGCTCTCCTT-3'
Ifnα4	NM_010504	.: 5'-TTCTGCAATGACCTCCATCA-3'	.: 5'-TATGTCCTCACAGCCAGCAG-3'
Ifnα6	NM_206871	.: 5'-TGGAATGCAACCCTCCTAGA-3'	.: 5'-TCAGGGGAAGTGCCTGTATC-3'
Ifnβ	NM_010510	.: 5'-CTCAGGGTGTCGATGAGGTC-3'	.: 5'-CCCAGTGCTGGAGAAATTGT-3'
Ifnγ	NM_008337	.: 5'-ACAGCAAGGCGAAAAAGGAT-3'	.: 5'-TGAGCTCATTGAATGCTTGG-3'
Il-2	NM_008366	.: 5'-GCGGCATGTTCTGGATTTGACTC-3'	.: 5'-CCACCACAGTTGCTGACTCATC-3'
Il-4	NM_021283	.: 5'-ATGGATGTGCCAAACGTCCT-3'	.: 5'-AGCTTATCGATGAATCCAGGCA-3'
Il-5	NM_010558	.: 5'-GATGAGGCTTCCTGTCCCTACT-3'	.: 5'-TGACAGGTTTTGGAATAGCATTTCC-3'
Il-6	NM_031168	.: 5'-CTCTGCAAGAGACTTCCATCCA-3'	.: 5'-CTGCAAGTGCATCATCGTTGT-3'
Il-7	NM_008371	.: 5'-CAGGAACTGATAGTAATTGCCCG-3'	.: 5'-CTTCAACTTGCGAGCAGCACGA-3'
Il-9	NM_008373	.: 5'-TCCACCGTCAAAATGCAGCTGC-3'	.: 5'-CCGATGGAAAACAGGCAAGAGTC-3'
Il-10	NM_010548	.: 5'-ATCGATTTCTCCCCTGTGAA-3'	.: 5'-TGTCAAATTCATTCATGGCCT-3'
Il-15	NM_001254747	.: 5'-GTAGGTCTCCCTAAAACAGAGGC-3'	.: 5'-TCCAGGAGAAAGCAGTTCATTGC-3'
Il-23	NM_031252	.: 5'-CATGCTAGCCTGGAACGCACAT-3'	.: 5'-ACTGGCTGTTGTCCTTGAGTCC-3'
Ki67	NM_001081117	.: 5'-GAAGTCAAAGAGCAAGAGGCAA-3'	.: 5'-TCTTGAGGCTCGCCTTGATG-3'
Mx1	NR_003520	.: 5'-TCTGAGGAGAGCCAGACGAT-3'	.: 5'-CTCAGGGTGTCGATGAGGTC-3'
Pax5	NM_008782	.: 5'-TGACGCAGGTGTCATCGGTGAG-3'	.: 5'-ATTCGGCACTGGAGACTCCTGA
Pcna	NM_011045	.: 5'-TGGATAAAGAAGAGGAGGCG-3'	.:5'-GGAGACAGTGGAGTGGCTTT-3'
Prdm1	NM_007548	.: 5'-ACCAAGGAACCTGCTTTTCA-3'	.: 5'-TAGACTTCACCGATGAGGGG-3'
Rorc	NM_001293734	.: 5'-ACAGAGACACCACCGGACAT -3'	.: 5'-GGTGATAACCCCGTAGTGGA -3'
Tbx21/Tbet	NM_019507	.:5'-TCAACCAGCACCAGACAGAG -3'	.: 5'-ATCCTGTAATGGCTTGTGGG -3'
Tgfβ	NM_011577	.: 5'-GGAGAGCCCTGGATACCAAC-3'	.: 5'-CAACCCAGGTCCTTCCTAAA-3'
Tlr7	NM_001290755	.: 5'-GGATGATCCTGGCCTATCTC-3'	.: 5'-TGTCTCTTCCGTGTCCACAT-3'
Tlr9	NM_031178	.: 5'-CAGTTTGTCAGAGGGAGCCT-3'	.: 5'-CTGTACCAGGAGGGACAAGG-3'
TNF	NM_013693	.: 5'-GATCGGTCCCCAAAGGGATG-3'	.: 5'-GGTGGTTTGCTACGACGTG-3'
Tnfrsf13b/Taci	NM_021349	.: 5'-GGTCAGACAACTCAGGAAGGCA-3'	.: 5'-CCAAGAAACAGCAGAAGATGGCG-3'
Tnfrsf13c/Bcmd	NM_028075	.: 5'-CACTTCAGAAGGAGTCCAGCAAG-3'	.: 5'-CTGTCTGCATCTTCTTTGAGCGG-3'
Tnfrsf17/Bcma	NM_011608	.: 5'-GCAACCTGTCAGCCTTACTGTG-3'	.: 5'-GTGCCAAAGAGAGGACCAAGGT-3'
Tnfsf13/April	NM_001159505	.: 5'-GTTGCTCTTTGGTTGAGTTGGG-3'	.: 5'-GTTGGATCAGTAGTGCGACAGC-3'
Tnfsf13b/Baff	NM_033622	.: 5'-CCTCCAAGGCATTTCCTCTT-3'	.: 5'-GACTGTCTGCAGCTGATTGC-3'
Xbp1	NM_013842	.: 5'-TAGACCTCTGGGAGTTCCTCCA-3'	.: 5'-TGGACTCTGACACTGTTGCCTC-3'
Zc3h12a/Mcpip1	NM_153159	.: 5'-CCTGTGGTCATCGACGGAAG-3'	.: 5'-GAAGGATGTGCTGGTCTGTGATA-3'

Disease Models & Mechanisms • Supplementary information
